# A neuroanatomically grounded Hebbian-learning model of attention–language interactions in the human brain

**DOI:** 10.1111/j.1460-9568.2008.06015.x

**Published:** 2008-01

**Authors:** Max Garagnani, Thomas Wennekers, Friedemann Pulvermüller

**Affiliations:** 1 MRC Cognition & Brain Sciences Unit 15 Chaucer Road, Cambridge CB2 7EF, UK; 2 Centre for Theoretical and Computational Neuroscience, University of Plymouth Plymouth, UK

**Keywords:** electroencephalography, magnetoencephalography, neural networks, perisylvian cortex, simulation, synaptic plasticity

## Abstract

Meaningful familiar stimuli and senseless unknown materials lead to different patterns of brain activation. A late major neurophysiological response indexing ‘sense’ is the negative component of event-related potential peaking at around 400 ms (N400), an event-related potential that emerges in attention-demanding tasks and is larger for senseless materials (e.g. meaningless pseudowords) than for matched meaningful stimuli (words). However, the mismatch negativity (latency 100–250 ms), an early automatic brain response elicited under distraction, is larger to words than to pseudowords, thus exhibiting the opposite pattern to that seen for the N400. So far, no theoretical account has been able to reconcile and explain these findings by means of a single, mechanistic neural model. We implemented a neuroanatomically grounded neural network model of the left perisylvian language cortex and simulated: (i) brain processes of early language acquisition and (ii) cortical responses to familiar word and senseless pseudoword stimuli. We found that variation of the area-specific inhibition (the model correlate of attention) modulated the simulated brain response to words and pseudowords, producing either an N400- or a mismatch negativity-like response depending on the amount of inhibition (i.e. available attentional resources). Our model: (i) provides a unifying explanatory account, at cortical level, of experimental observations that, so far, had not been given a coherent interpretation within a single framework; (ii) demonstrates the viability of purely Hebbian, associative learning in a multilayered neural network architecture; and (iii) makes clear predictions on the effects of attention on latency and magnitude of event-related potentials to lexical items. Such predictions have been confirmed by recent experimental evidence.

## 1. Introduction

**Fig. 1:**
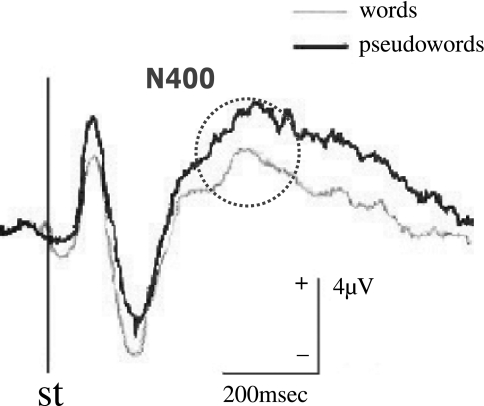
Event-related potential responses to spoken words (light grey line) and pseudowords (dark grey line) averaged across 14 subjects, as recorded from scalp electrode Cz. The vertical line labelled ‘st’ indicates stimulus onset time. The labelled dashed circle highlights the difference between negative component of event-related potentials peaking at around 400 ms (N400) [adapted from Sinai & Pratt (2002)].

Our brains store knowledge about common activities, objects, faces and words as distributed memory circuits. Using neurophysiological techniques, it should be possible to reveal (i) the presence and full activation (‘ignition’) of these ‘memory traces’ when previously learned patterns appear in the sensory input, and (ii) the absence of their activation when meaningless, unfamiliar stimuli are presented. Experimental evidence obtained using electroencephalography (EEG) and magnetoencephalography (MEG) indeed revealed different patterns of brain activation for meaningful material and senseless unknown stimuli. The major brain response indexing ‘sense’ is the N400, a negative-going event-related potential (ERP) peaking around 400 ms after stimulus onset (Kutas & Hillyard, 1980). The N400 is larger for senseless materials (e.g. meaningless pseudowords, semantically incoherent text) than for matched meaningful language (common words or coherent text) and is elicited under conditions where subjects are attending to the input (Kutas & Hillyard, 1980; Barber & Kutas, 2007). Figure 1 shows an example of this type of ERP response (adapted from Sinai & Pratt, 2002). The study consisted of an auditory lexical decision task with words and pseudowords. The data plotted here are the grand averages of the ERP responses to stimuli to which no response was requested.

**Fig. 2:**
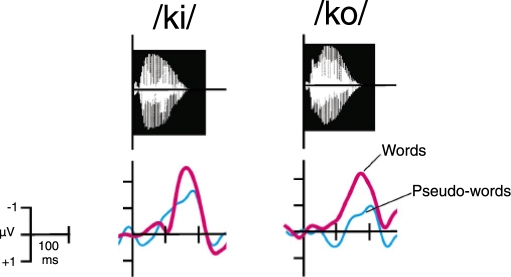
Mismatch negativities (MMNs) elicited by the critical syllables /ki/ (left) and /ko/ (right) in native Finnish speakers when placed in a word context (red traces) and in a pseudo-word context (blue). Each MMN curve is calculated as the difference between the event-related potentials elicited by the same sound when presented as a deviant or as a frequent standard. The acoustic waveforms of the stimuli which elicited the MMNs are shown at the top [adapted from Pulvermüller *et al.* (2001)].

More recently, short-latency MEG and EEG differences in the brain response to words and pseudowords have also been recorded, e.g. in the mismatch negativity (MMN) brain response. The MMN is an early pre-attentive event-related response (latency 100–250 ms) elicited by infrequent acoustic events (so-called ‘deviant stimuli’) occasionally occurring among frequently repeated sounds (‘standard stimuli’) (Näätänen *et al.*, 1978). The MMN is elicited even when subjects are heavily distracted and, in this case, is larger for words than for pseudowords, thus exhibiting the reverse pattern to that seen for the N400 (Korpilahti *et al.*, 2001; Pulvermüller *et al.*, 2001; Shtyrov & Pulvermüller, 2002; Pettigrew *et al.*, 2004; Pulvermüller & Shtyrov, 2006). Figure 2 shows the MMNs obtained from ERPs of native speakers of Finnish to word and pseudoword stimuli (from Pulvermüller *et al.*, 2001).

The question of why these brain indicators of lexico-semantic processes arise at different latencies and present opposite relative magnitude is, as yet, unanswered. A possible explanation may be that words and pseudoword stimuli are being processed in different testing conditions. In particular, different tasks (such as lexical decision or passive listening under distraction) imply different levels of attention, and attentional load might modulate the brain responses to words and pseudowords differentially. A number of studies have confirmed that ERPs and MMN amplitudes are modulated by the attentional load that is required by the task under which they are elicited (Alho *et al.*, 1992; Woods *et al.*, 1992); Otten *et al.*, 1993; Bentin *et al.*, 1995; Woldorff *et al.*, 1998; Pulvermüller, 2007). Indeed, Szymanski *et al.* (1999) reported that ‘top-down controls not only affect the amplitude of the MMN, but can reverse the pattern of MMN amplitudes among different stimuli’. However, these studies fail to provide an account, at the brain circuit level, of the mechanisms that underlie the differential neurophysiological responses to words and pseudowords. How do the different neural processes interact so as to produce brain responses having opposite magnitude and different latencies?

One way to address this question is to develop a computational model at the level of nerve-cell circuits that can reproduce spatial and temporal aspects of brain activity in the relevant cortical areas and provide a mechanistic explanation of the existing neurophysiological findings. We implemented a brain-inspired neural network that models neuroanatomical, connectivity and neurophysiological properties of the left hemisphere language areas that are situated close to the sylvian fissure (perisylvian cortex, here referred to as ‘language cortex’), and used it to simulate and explain, at the cortical-circuit level, (i) brain processes of early word learning and (ii) the effects of lexicality (the processing difference between words and pseudowords) and attentional load on the processing of speech and language.

## 2. Background

This section provides the theoretical background and motivation for the model implemented (described in detail in Section 3). We introduce the cognitive constructs of interest, identify the underlying neuroanatomical structures and neural mechanisms, and characterize the mapping between such brain mechanisms and corresponding entities of the model.

### 2.1. Language, learning and word-related neuronal circuits

In cognitive terms, the main objects of interest of this study are the building blocks of language, i.e. words. We start from the hypothesis that the neural correlate of a word is a memory circuit (‘trace’) that develops during early language acquisition (Pulvermüller, 1999). It is well known that, even during the earliest stage of speech-like behaviour (Fry, 1966), near-simultaneous correlated activity is present in different brain parts, especially those areas controlling speech output (left inferior prefrontal cortex) and those where neurones respond to auditory features of speech (left superior temporal lobe). In the adult brain these areas are reciprocally connected (see Section 2.2). We conjecture that, through Hebbian learning mechanisms (Hebb, 1949), such connections allow the acquisition of sensory–motor associations between co-occurring cortical patterns of activity, in such a way that, e.g. listening to speech sounds involving specific articulators leads to the ‘lighting up’ of the corresponding motor representations. A significant body of experimental evidence confirms the presence of speech–motor associations as networks of strongly interconnected neurones distributed between left superior temporal and inferior frontal cortex (Zatorre *et al.*, 1996; Pulvermüller, 1999; Pulvermüller *et al.*, 2001; Fadiga *et al.*, 2002; Watkins *et al.*, 2003; Watkins & Paus, 2004; Wilson *et al.*, 2004; Pulvermüller & Shtyrov, 2006), and their role in language processing. We refer to such distributed networks of strongly and reciprocally connected neurones as to ‘Hebbian neuronal circuits’ (HNCs) or cell assemblies (Hebb, 1949; Braitenberg, 1978; Palm, 1982; Wennekers *et al.*, 2003). An HNC can be thought of as a highly specialized functional unit that ‘responds’ by becoming fully active only when a specific brain activation pattern − brought about by the sensory (or internal) stimulation − conveys at least a critical amount of activation in its neuronal circuits. Sensory–motor HNCs could receive their input (e.g. lexical items, words) either through the auditory or the motor modalities.

**Fig. 3:**
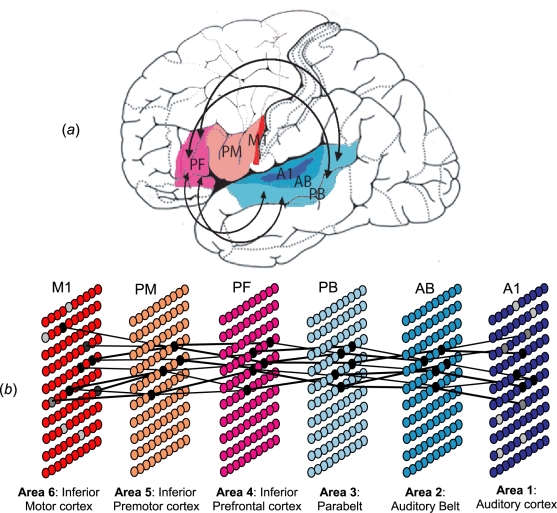
The relevant areas of the perisylvian cortex, overall network architecture and mapping between the two. (a) The six different areas of the perisylvian language cortex modelled, labelled as M1, PM, PF, A1, AB and PB, and indicated in different colours. Black arrows indicate long-distance cortico-cortical connections between the auditory and motor association areas (see text for details). (b) The six-area network model and an illustration of the type of distributed functional circuit (Hebbian neuronal circuit or cell assembly) that developed during learning of perception-action patterns. The colours and labels indicate the mapping between each model area and the respective cortical area that it represents. Each small filled oval represents an excitatory neuronal pool; thick and thin lines indicate, respectively, strong reciprocal and weak (and/or non-reciprocal) connections. Coactivated cells are depicted as black (or grey) ovals. Only forward and backward links between coactivated cells are shown. Inhibitory interneurones are not depicted [adapted from Garagnani *et al.* (2007)].

We simulated the setting up of such sensory–motor links for lexical items at early stages of language acquisition in a neural network model of the left-perisylvian language cortex. To induce HNC formation in the model, we repeatedly exposed the network to pre-determined pairs of (random and sparse) activation configurations, each activation-pattern pair representing the model equivalent of an auditory-articulatory word form, and allowed the network's synaptic weights to adapt through Hebbian-type learning (see below and Section 3.1.2). [Although a complex network structure (six layers, see Fig. 3) approximating the neuroanatomy of the language cortex was implemented, no computational ‘tricks’ such as back-propagation of errors were applied; we restricted learning to biologically established Hebbian algorithms.] Our main prediction was that well-defined, strongly connected HNCs would develop for the sensory–motor pairs, representing the network equivalents of brain circuits for words (Pulvermüller, 2003; Garagnani *et al.*, 2007). Due to their strong internal and reciprocal connections, HNCs were also expected to exhibit ‘memory’ and ‘pattern completion’ features (see also Wennekers & Palm, 2007), i.e. reverberation of excitation within the circuit in the absence of any input following stimulation and full activation after only partial stimulation. The method adopted to test these predictions and the results obtained are described in Sections 3.2 and 4.1, respectively.

We postulate that the brain mechanisms mediating the development of specialized HNCs (driven by the repeated presentation of the same sensory–motor input patterns) are generic Hebbian mechanisms of associative learning, and take the phenomena of long-term potentiation (LTP) and long-term depression (LTD) to be the neural correlates of learning. LTP and LTD consist of a long-term increase or decrease in synaptic strength resulting from pairing pre-synaptic activity with specific levels of post-synaptic membrane potentials (Buonomano & Merzenich, 1998; Malenka & Nicoll, 1999). These phenomena are believed to play a key role in experience-dependent plasticity, memory and learning (Rioult-Pedotti *et al.*, 2000; Malenka & Bear, 2004). In the present model, we implemented synaptic plasticity by allowing the strength (weight) of the connections between different cells to adapt according to a mathematical specification of LTP/LTD, based on the Artola-Bröcher-Singer (ABS) rule (Artola & Singer, 1993) (see Section 3.1.2 for details).

Finally, in the attempt to replicate and explain the effects of lexicality and attention on the processing of speech, we used the resulting trained network to simulate the response of the language cortex to words and pseudowords under variable attentional load. The details of the methods adopted for this part of the study and corresponding results are presented in Sections 3.3 and 4.2, respectively.

### 2.2. The language cortex

In this section we specify the relevant cortical areas involved in language processing that we reproduced in the model, their cortical loci and connectivity features. Some of the structural features are evident from investigations of the human brain; however, others, especially the fine-grained wiring between and within cortical areas, have to be inferred from monkey studies (Pulvermüller, 1992, 1999).

The primary cortices involved in spoken language processing include (i) the primary auditory area [Brodman's area (BA) 41], located in the caudal part of the planum supratemporale (the part of the upper convolution of the temporal lobe that lies in the sylvian fissure), and (ii) the ventral part of the primary motor cortex (BA 4), situated near the sylvian fissure (Pulvermüller, 1992, 1999). These two areas are active during perception of speech sounds and execution of articulatory movements, respectively (see areas ‘A1’ and ‘M1’ in Fig. 3a). The third primary cortex involved in spoken language processing is the somatosensory cortex, located posterior to the central sulcus; in particular, this includes the inferior parts of BA 1, 2 and 3, which are necessary for sensations within the mouth region. In both the primary auditory and somatosensory areas, afferent fibres carrying sensory input enter the cortex; the primary motor cortex, however, contains large pyramidal cells that project to motor neurones controlling articulatory muscles.

According to neuroanatomical studies in the rhesus monkey (*Macaca mulatta*) the primary perisylvian motor or articulatory cortex is tightly connected to the premotor (secondary) regions anterior to it. These, in turn, are connected to region around the inferior branch of the arcuate sulcus (Pandya & Yeterian, 1985), in the inferior prefrontal cortex. Experimental evidence (Fuster, 1997; Rizzolatti *et al.*, 2001) suggests that similar connection patterns are likely to be present in the homologous structures in man, located in the ventral motor (BA 4) and premotor (BA 6) cortices, and within BA 44 and BA 45 (Broca's area) (these areas are labelled ‘M1’, ‘PM’ and ‘PF’ in Fig. 3a for primary motor, premotor and prefrontal cortex, respectively).

As discussed in detail in Pulvermüller (1992), a similar picture can be drawn for the somatosensory and auditory cortex (e.g. see Kaas & Hackett, 2000; Rauschecker & Tian, 2000; Scott *et al.*, 2000), i.e. each of the primary cortices relevant for spoken language is strongly and reciprocally connected to its adjacent secondary region, which, in turn, is connected to its neighbouring association area. In the macaca, the relevant auditory areas are sometimes defined as ‘auditory core’, ‘belt’ and ‘parabelt’, respectively. These structures may be related (although an exact homology is not likely) to BA 41, 42 and 22 in the human brain (labelled ‘A1’, ‘AB’ and ‘PB’ in Fig. 3a for primary auditory, auditory belt and parabelt, respectively).

Studies in non-human (Pandya & Yeterian, 1985; Romanski *et al.*, 1999; Petrides & Pandya, 2002) and human (Makris *et al.*, 1999; Catani *et al.*, 2005) primates suggest that the respective association cortices of each of these primary areas are strongly and reciprocally interconnected with each other via the arcuate and uncinate fascicles, and the extreme capsule. These long-range cortico-cortical connections are indicated schematically in Fig. 3a by black ‘dorsal’ and ‘ventral’ arrow-pointed arcs.

The overall architecture of the neural network (see Fig. 3b) replicates the neuroanatomical features summarized here and interconnections of the language cortex. In particular, the model reproduces the main input cortical areas [the primary auditory cortex A1 and its surrounding belt and parabelt areas (AB and PB)] and the motor ‘output’ areas [the perisylvian motor cortex (M1) and areas PM and PF]. Each of these cortical areas is modelled as a 25 × 25 area of artificial (excitatory and inhibitory) cells (see Section 3.1 for details).

In addition to the six areas of excitatory/inhibitory cells, the network is endowed with a self-regulation mechanism (not shown in Fig. 3), necessary to maintain the total activity of the network within certain limits. It has been argued that the cortex must have developed a self-regulatory mechanism designed to keep activation between certain bounds (Braitenberg, 1978; Braitenberg & Schüz, 1998). Although there is agreement that the regulation of cortical activity is necessary, the exact characteristics of such a mechanism and the brain systems that realize it are still a matter of debate (see Fuster, 1995; Wickens, 1993; Pulvermüller, 2003, pp.78–81). In our model, the regulation mechanism is implemented as area-specific feedback loops that inhibit all cells of one area by the same amount, proportional to the total activity within that area (see Section 3.1.3 for details). In the brain, these circuits could be implemented by cortico-striato-thalamic loops (Miller & Wickens, 1991; Wickens, 1993). Finally, it should be noted that area-specific feedback inhibition (FI) not only provides the network with more stability but, as shown by theoretical work (Willshaw *et al.*, 1969; Braitenberg, 1978; Hopfield, 1982; Palm, 1982, 1987), can also solve the superposition problem, which requires the simultaneous full activation of two different HNCs to be prevented (Knoblauch & Palm, 2002).

### 2.3. Attention

As mentioned, attentional load modulating the brain responses to words and pseudowords differentially (e.g. Szymanski *et al.*, 1999) does not provide, in itself, a mechanistic explanation, at the brain-circuit level, of the neural causes underlying the different latencies and relative magnitudes found for the N400 and MMN responses. In order to explain such effects in terms of cognitive processes using a neural network model, it is necessary to provide a mapping of the relevant cognitive constructs (especially attentional load and lexical access) onto corresponding states and processes of the neurocomputational model.

Attention is a central theme in cognitive sciences (e.g. see Raz & Buhle, 2006 for a recent review). Selective attention is normally associated with the cognitive ability to internally focus on, or be aware of, only a small sub-set of the sensory information in input, relevant to current thought or behaviour, at the expense of the rest (Duncan, 1995, 2006). The ‘biased competition’ model of attention (Duncan, 1980, 1996; Duncan & Humphreys, 1989; Desimone & Duncan, 1995) proposes that different item representations (encoded by different populations of simultaneously active cortical neurones) compete, in a winner-take-all fashion, for the allocation of shared attentional resources, while a top-down signal biases this competition so that the stimulus relevant to the current task or behaviour ‘wins’ (Duncan, 2006). The winner-take-all mechanism may be implemented by assuming the existence of mutually inhibitory connections between different populations of strongly interconnected cells (HNCs). Crucially, the winner-take-all mechanism, postulated to be at the basis of (bottom-up) attentional selection processes, can also be realized via the cortical activity regulation mechanism, which is independently motivated by the need for functional stability (Braitenberg, 1978; Wickens, 1993; Fuster, 1995).

In our model, the response of the area-specific inhibitory loops (introduced in the last section) depends on (i) the strength of the connections forming such loops (henceforth called FI) and (ii) the level of activation present in the inhibitory loops at the time of sensory stimulation. We can now say that strong FI, leading to strong mutual inhibition between coactive HNCs, corresponds, at the cognitive level, to small amounts of attentional resources available for processing new sensory input (as small amounts of resources, or low attention, imply a tougher competition between costimulated lexical representations, and between ongoing activity in the brain and sensory input). In contrast, reduced FI (i.e. less competition between coactive HNCs) implements great availability of attentional resources; in this situation, more than one representation can be active at the same time (allowing phenomena like that of ‘divided attention’ or attention to a large perceptual space).

The use of non-specific inhibition to successfully model different aspects of attention is not a new idea (Szabo *et al.*, 2004; Rolls & Deco, 2002; Deco *et al.*, 2004). However, unlike previous studies (Deco *et al.*, 2004; Deco & Rolls, 2005), we do not attempt to model here the top-down attentional signal responsible for ‘biasing’ the competition amongst the coactive HNCs and, thus, selecting which item representations ultimately enter the attentional focus. Following Occam's razor, we decided not to make assumptions on such biasing signal; as it turns out, this feature was indeed unnecessary for the model to be able to simulate and explain the phenomema of interest here.

## 3. Methods

In this section, we provide a detailed description of the computational model implemented (Section 3.1) and of the simulations carried out (Sections 3.2 and 3.3). Our approach follows similar attempts to build models linking neuronal circuits to functional systems, especially in the domain of visual and auditory processing (Tagamets & Horwitz, 1998; Corchs & Deco, 2002; Westermann & Miranda, 2002, 2004; Husain *et al.*, 2004; Deco & Rolls, 2005; Guenther *et al.*, 2006). The features that set apart the present model from these and other connectionist models will be discussed in Section 5.

### 3.1. Network structure and function

A complete characterization of the computational model requires describing both the fine-grained (or neuronal) and high (or systems) level. For each of these levels, the structure (the sub-components and how they are integrated) and function (the result of the dynamic interactions of the component parts) will be explained. In the three following sub-sections, we start from the basic computational unit of our model (the ‘cell’, representing a local pool of neurones) and move on to the higher levels of area and network (a ‘system’ of cortical areas), alternating structural and functional descriptions as appropriate.

The main quality criterion for the model was biological realism. This was broken down into systems-level realism (especially the anatomical and connectivity features of the model, linking it to a specific brain part − the perisylvian cortex) and microphysiological- level realism. Bearing this criterion in mind, it was necessary to find a good compromise between the two conflicting additional goals of developing a model that was sufficiently realistic so as to allow the emergence of the relevant complex processes observed in the human brain, and sufficiently simple so as to be computationally tractable. We achieved the latter by implementing a relatively simple (computationally speaking) ‘activity-regulation’ mechanism mimicking a coarse-grained attentional threshold control system (see Section 3.1.3) and by keeping the total number of cells in the network within a manageable range.

#### 3.1.1. Model of cortical neurones

The basic computational unit of our model is the ‘cell’, a neurone-like element whose dynamic behaviour and response are based on those of real neurones. We do not aim at simulating individual cortical neurones but rather employ a lumped or mean-field type model in the simulations, where each cell represents the average activity of a local pool of neurones or ‘column’ (Wilson & Cowan, 1973; Eggert & van Hemmen, 2000). Analogous approaches based on the neuronal mass model (Nunez, 1974; Freeman, 1978) have been used in the past as generative models of EEG/MEG and functional magnetic resonance imaging (fMRI) signals (David & Friston, 2003; Husain *et al.*, 2004).

In our model, each cell or ‘node’ of the network may be considered to represent a cortical column of approximately 0.25 mm^2^ size (Hubel, 1995; Mountcastle, 1997), containing ∼2.5 × 10^4^ neurones (Rockel *et al.*, 1980; Braitenberg & Schüz, 1998, p. 25). [These figures are meant to provide only an estimate of the grain of the model; as noted in Hubel (1995), the size of a macrocolumn (or ‘module’) varies substantially between cortical layers (going from 0.1 mm^2^ in layer 4C to 4 mm^2^ in layer 3) and cortical areas (their p. 130).] Each cell has a membrane potential *V*(*x*,*t*) (reflecting temporal low-pass properties of local neurone pools, see Eqn 1 below) and transforms its potential into firing rate by means of a sigmoid output function (Eqn 2) reflecting local firing activity. The membrane potential *V*(*x*,*t*) at time *t* of a model cell *x* with membrane time constant *τ* is governed by the equation:
(1)
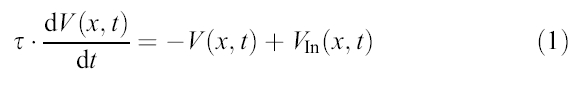

where *V*_In_(*x*,*t*) is the total input to cell *x*, representing the sum of all excitatory and inhibitory post-synaptic potentials acting upon neurone pool *x* at time *t* (inhibitory inputs are given a negative sign); these sub-synaptic excitatory and inhibitory post-synaptic potentials drive inward currents in neurones of pool *x*, producing the charging of their somata.

The value *O*(*x*,*t*) produced as output by a cell *x* is the only signal propagated by *x* to other cells. The output value *O*(*x*,*t*) of a cell *x* at time *t* is a piecewise linear sigmoid function of the cell's membrane potential *V*(*x*,*t*): (2)
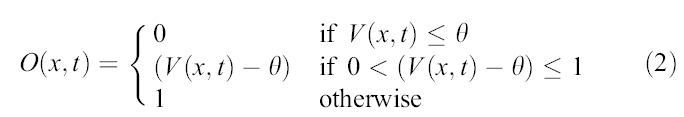


In other words, the output is clipped into the range [0,1] and has slope 1 between the lower and upper thresholds *θ* and *θ* + 1. The value of *θ* is initialized to 0 but varies in time (see below). The output value of a cell *x* at time-step *t* represents the cumulative (graded) output (number of action potentials per time unit) of cluster *x* at time *t*; this value predicts action potential frequency in a certain time-window (centred on *t*) and, thus, changes in the post-synaptic potentials induced by the neurone pool *x* in all of the synapses downstream from it.

We integrate the low-pass dynamics of the network cells (Eqn 1) using the Euler scheme with step size Δ*t* (Press *et al.*, 1992). The values for Δ*t* chosen in the simulations was 0.5 (in arbitrary units of time). A relatively wide integration step size was chosen to speed up simulations of the full model, as for the time-continuous (non-spiking) neurone model considered here, smaller step-sizes lead to largely the same network properties. An estimate of the ‘real’ duration of one simulation step (Δ*t*) can be obtained by matching the simulated neurophysiological responses with the corresponding experimental data. According to such approximate mapping (see Section 5.3.1 for details), one Δ*t* is equivalent to about 20 ms.

Cells come in two different types: excitatory cells (called ‘E-cells’) and inhibitory cells (or ‘I-cells’); they model populations of cortical pyramidal neurones and pools of inhibitory interneurones, respectively. The behaviour of an E-cell is specified entirely by Eqns 1 and 2. I-cells behave identically, except that their output *O*(*x*,*t*) does not saturate at high values [i.e. it is simply *V*(*x*,*t*) for *V*(*x*,*t*) > 0 and 0 elsewhere]. In addition, the value used for the time constant *τ* in Eqn 2 is 2.5 for E-cells and 5 for I-cells (in simulation time-steps or Δ*t*'s). The use of these two different values is motivated by the higher time constants of inhibitory post-synaptic potentials as compared with excitatory post-synaptic potentials (Kandel *et al.*, 2000, p. 923). Assuming that Δ*t*∼20 ms, E- and I-cells have time constants of about 50 and 100 ms, respectively. Notice, however, that these values should not be interpreted as model correlates of inhibitory and excitatory post-synaptic potential time constants, as each cell here represents a population of neurones.

Cells can be connected by links (‘synapses’). Each synapse is associated to a numeric value (weight) representing the efficacy of that connection. If cell *x* is linked to cell *y* with weight *w*_*x*,*y*_, it contributes a potential *O*(*x*,*t*)· *w*_*x*,*y*_ to the total input *V*_In_(*y*,*t*) of the target cell *y*, where *O*(*x*,*t*) is defined by Eqn 2. Without loss of generality, we limit the numeric values of the weights to the range [0,1].

Finally, E-cells are also endowed with a simple mechanism of adaptation. When a real neurone receives above-threshold stimulation and starts firing, it produces a few spikes at high frequency; if the stimulus is maintained, the rate gradually gets lower and then levels off. This phenomenon is normally referred to as neural (or ‘spike-rate’) adaptation (Kandel *et al.*, 2000, p. 424; Brenner *et al.*, 2000). In the model, adaptation is realized (in E-cells only) by allowing the value of parameter *θ* in Eqn 2 to vary in time. In particular, *θ* is tied to the time-average of the cell's recent output [for computational efficiency, the time-average of the output *O*(*x*,*t*) of each E-cell is estimated numerically by low-pass filtering *O*(*x*,*t*) with adaptation time constant *τ*_A_ = 15. The final *θ* is then obtained by scaling down the estimated time-average by a small factor (0.026 in our simulations; see Appendix A).] so that higher-(lower-)than-average values of *O*(*x*,*t*) lead to a gradual increase (decrease) in *θ*. This has the effect of adapting the cell's response to the input level.

#### 3.1.2. Modelling Hebbian synaptic plasticity

The weights of the links between E-cells are not fixed but are allowed to change in time, modelling the neurobiological phenomena of LTP and LTD (Buonomano & Merzenich, 1998; Malenka & Nicoll, 1999). The computational abstraction of LTP and LTD implemented here is based on the ABS rule (Artola & Singer, 1993), an extended and more neurobiologically accurate version of the well-known ‘Bienenstock-Cooper-Munro’ (BCM) rule (Bienenstock *et al.*, 1982). Whereas the BCM rule had been originally developed to account for cortical organization and receptive field properties during development, the ABS rule is derived from neurophysiological data obtained in the mature cortex. Such experimental data suggest that similar pre-synaptic activity (i.e. brief activation of an excitatory pathway) can lead to synaptic LTD or LTP, depending on the level of post-synaptic depolarization co-occurring with the pre-synaptic activity. In particular, data from structures susceptible to both LTP and LTD (Artola *et al.*, 1990) suggest that a stronger depolarization is required to induce LTP than to initiate LTD. [The level of post-synaptic depolarization determines the amount of Ca^2+^ entering the dendritic spine; a moderate rise in Ca^2+^ leads to a predominant activation of phosphatases and LTD, whereas a stronger increase favours activation of kinases and LTP.] Accordingly, the ABS rule postulates the existence of two voltage-dependent thresholds in the post-synaptic cell, called *θ*_–_ and *θ*_+_ (with *θ*_–_ < *θ*_+_). The direction of change in synaptic efficacy depends on the membrane potential of the post-synaptic cell. If the potential reaches the first threshold (*θ*_–_), all active synapses depress; if the second threshold (*θ*_+_) is reached, all active synapses potentiate. Unlike in the BCM rule (where LTD takes place even with very small post-synaptic potentials), if post-synaptic depolarization remains below *θ*_–_, then the synaptic efficacy of all synapses remains unchanged, regardless of any pre-synaptic activity.

We implemented a tractable version of the full ABS model (Artola & Singer, 1993), as described below. The simplifications involve discretizing the continuous range of possible synaptic efficacy changes to only two levels, +Δ*w* and –Δ*w* (where Δ*w* ∈ [0,1] is a fixed quantity <<1 representing the learning rate), and defining as ‘active’ at time *t* any input link from a cell *x* such that *O*(*x*,*t*) > *θ*_*pre*_, where *θ*_*pre*_ ∈ [0,1] is an arbitrarily fixed threshold representing the minimum level of pre-synaptic activity required for LTP to occur. More precisely, given any two E-cells *x* and *y* currently linked with weight *w*_*t*_(*x*,*y*), the new weight *w*_*t*+1_(*x*,*y*) is calculated as follows: (3)
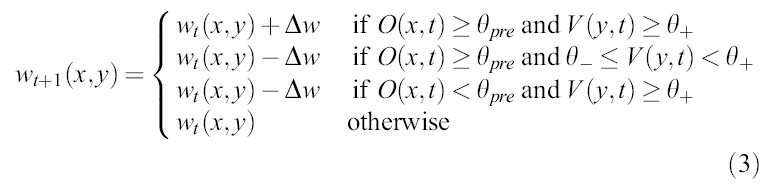
 where *V*(*y*,*t*) is the membrane potential of the post-synaptic cell *y* at time *t* (Eqn 2). In our simulations, we used *θ*_–_ = 0.15, *θ*_+_ = 0.25, *θ*_*pre*_ = 0.05 and Δ*w* = 0.0005. The three cases of Eqn 3 model, respectively: (i) homosynaptic and associative LTP; (ii) homosynaptic LTD; and (iii) heterosynaptic LTD. The latter type of LTD involves synaptic change at inputs that are themselves inactive but that undergo depression due to depolarization spreading from adjacent active synapses. This form of LTD has been observed in the hippocampus and neocortex (Hirsch *et al.*, 1992); the induction protocols require strong post-synaptic activation (e.g. high-frequency stimulation of the cell through excitatory inputs), which is reflected in Eqn 3 by the condition requiring *V*(*y*,*t*) ≥ *θ*_+_.

#### 3.1.3. System-level architecture

**Fig. 4:**
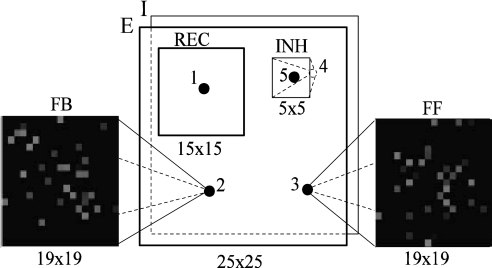
Connectivity and structure of a single ‘cortical’ area. Each model area comprises two overlaying bidimensional layers of 25 × 25 excitatory (E) and inhibitory (I) cells each. Each E-cell (depicted as a filled black circle) projects (in a sparse, ‘patchy’ manner) to neighbouring E-cells in the same area (REC, cell 1) but also to E-cells in the previous (FB) and next (FF) areas via feedback (cell 2) and feedforward (cell 3) connections, respectively. The small brighter squares on the black background represent an example of where such patchy links might be established, brighter levels of grey indicating stronger link weights. Inhibitory cells (e.g. I-cell 4, depicted as a dashed circle) receive input from (all) E-cells located within an overlying 5 × 5 neighbourhood (INH) and inhibit the E-cell located at the centre of it (i.e. I-cell 4 inhibits E-cell 5). Area-specific inhibition feedback loops are not depicted. See also Section 3.1.3.

The neural network model (see Fig. 3) reproduces the auditory input areas (A1, AB and PB) and motor output areas (M1, PM and PF) of the language cortex identified in Section 2.2. Each of these (primary, secondary and association) areas is modelled as a lattice (grid) of interconnected cells; more precisely, each model area consists of a layer of 25 × 25 graded-response E-cells sitting on an underlying layer of 25 × 25 graded-response I-cells (not shown in Fig. 3b). If we assume that each E-cell (together with its underlying I-cell) represents a cortical column of size 0.25 mm^2^, each model area simulates the activity of a cortical area of about 625 × 0.25 mm^2^ ≈ 1.6 cm^2^. Both between-area (cortico-cortical) and within-area (lateral and recurrent) excitatory connections are realized, so that one E-cell can project to neighbouring E-cells within the same area and to E-cells of adjacent areas. Links between non-adjacent areas are not implemented (however, note that the two adjacent Areas 3 and 4 correspond to cortical areas that are not anatomically adjacent). This results in a hierarchical architecture that closely reflects the neuroanatomical data described earlier; in fact, the primary cortical areas (M1 and A1) are reciprocally connected to their neighbouring secondary areas (PM and AB); these, in turn, are reciprocally linked to their respective association areas (PF and PB), which are also interconnected (via long-range cortico-cortical links). The same type of hierarchical (or multilayer) architecture is also found in other sensory modalities, a notable example being the visual system (Maunsell & Newsome, 1987; Lamme & Roelfsema, 2000; Young, 2000). Finally, the two layers of E- and I-cells that constitute a single area are closely and reciprocally connected, forming negative-feedback circuits that model local activity control and lateral inhibition (i.e. winner-take-all) mechanisms. The presence of lateral inhibition and next-neighbour connectivity, based on known characteristics of the cortex (Douglas & Martin, 2004; Braitenberg & Schüz, 1998), is shared by many neurobiologically based connectionist models of the cortex (e.g. Riesenhuber & Poggio, 1999; Rolls & Deco, 2002; Yuille & Geiger, 2003). The precise characteristics of the connections realized are now described in more detail (refer to Fig. 4).

The recurrent excitatory links projecting from an E-cell to E-cells of the same area are realized as follows: a link from a cell A to a cell B is created with probability *p*_link_(A,B), where *p*_link_(A,B) decreases as the cortical distance between A and B (in lattice units, i.e. cells) increases, according to a Gaussian curve. More formally: (4)
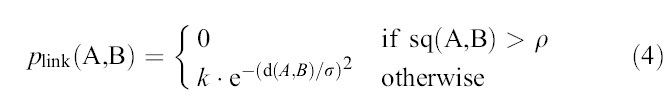
 where *ρ* ∈ ℵ^+^ (the set of positive integers), *σ* ∈ ℜ^+^(the set of positive real numbers), *k* ∈ [1,0], and, if cells A and B have lattice (or area) co-ordinates (*x*_A_,*y*_A_) (*x*_B_,*y*_B_), respectively, then sq(A,B) and d(A,B) are defined as (5)
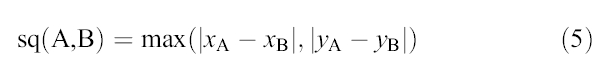
(6)
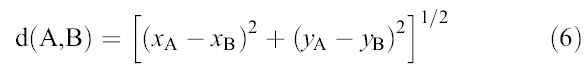


In short, if B is located outside a square of (2*ρ* + 1)^2^ cells centred on A, the probability of a ‘synapse’ being created between A and B is null; otherwise, the probability is a Gaussian function (with variance *σ*^2^ and amplitude *k*) of the (‘cortical’) Euclidean distance between cells A and B (we used *k* = 0.15, *ρ* = 7 and *σ* = 4.5). [From Eqn 4, it follows that the probability of having any E-cell linked to itself is exactly *k*.] Thus, E-cells that are more than *ρ* lattice units (cells) apart cannot be (directly) connected. If one cell is assumed to represent a cortical column of size ≈0.5 × 0.5 mm^2^, the radius of within-area lateral projections is 0.5 × *ρ* ≈ 3.5 mm. Finally, if an excitatory link between two E-cells is created, its weight is initialized to a real number chosen randomly between 0 and *w*_up_, with *w*_up_ = 0.1.

Excitatory ‘forward’ and ‘backward’ links, connecting any E-cell A with co-ordinates (*x*_A_,*y*_A_) in area *a*_1_ to other E-cells of an adjacent area, *a*_2_, are realized in the same way. Randomly weighted links may only be established between A and a square of (2*ρ* + 1)^2^ cells centred on cell (*x*_A_,*y*_A_) in area *a*_2_, where the probability of creating a link between any two cells is defined by Eqn 4. For forward and backward connections, the parameters used are *k* = 0.28, *ρ* = 9 and *σ* = 6.5. Hence, within-area projections are smaller and less dense, on average, than between-area projections (see Fig. 4). Although the exact values of these parameters were calibrated through simulation studies, the type of excitatory connections realized in the network is biologically motivated and aims at reproducing the next-neighbour, patchy and sparse connectivity typically found in the mammalian cortex (Gilbert & Wiesel, 1983; Amir *et al.*, 1993; Braitenberg & Schüz, 1998; Douglas & Martin, 2004). [The stimuli representing acoustic or motor cortical activity were also activating the network in a random and sparse way (see Section 3.2 for details). Some experimental evidence suggests that the neural code adopted by the brain to represent complex stimuli may indeed be sparse (e.g. Rolls & Tovee, 1995; cf. Reddy & Kanwisher, 2006 for discussion).]

The reciprocal connections between a layer of E-cells and its underlying lattice of inhibitory I-cells are similar but somewhat simpler than those described above. Firstly, each I-cell (pool of interneurones) receives excitatory inputs from all E-cells situated within an overlying 5 × 5 neighbourhood (i.e. within a radius *ρ* = 2, equivalent to ∼1 mm) and projects back (with weight = 1) to the single E-cell located directly above it. The smaller radius *ρ* reflects the fact that inhibitory interneurones (basket or chandelier cells) present smaller and more verticalized dendritic arborizations than pyramidal cells do (Jin *et al.*, 2001; Somogyi *et al.*, 1981). Moreover, the weight of the lateral connections is not assigned randomly but decreases with the distance according to the Gaussian function defined in Eqn 4 (with *σ* = 2.0, *k* = 0.295). This negative feedback circuit functions both as a ‘local’ activation control and lateral inhibition mechanism, simulating the action of a pool of inhibitory interneurones surrounding a pyramidal cell in the cortex (Braitenberg & Schüz, 1998).

In order to prevent overactivation, we implemented a self-regulation mechanism by introducing area-specific FI loops that control the total activity within each area. More precisely, all E-cells of each area project (with weight = 1) to a single, area-specific I-cell (not part of the underlying layer of ‘local’ I-cells), henceforth called FI-cell. Each FI-cell projects back to all of the E-cells of that area, providing an amount of inhibition proportional to the total activity within that area. As explained in Section 2.3, the strength (weight) of these FI loops can be varied and used to simulate the presence of different amounts of attentional resources during language processing.

A complete formulation of the computational features of the model, summarizing and integrating the description given in this section, is reported in Appendix A.

### 3.2. Learning of HNCs

The neural network model of the left-perisylvian language cortex described in the previous section was used to simulate brain processes of early language acquisition. The process of Hebbian association between sensory–motor patterns (see Section 2.1) was simulated in the network through repeated simultaneous activation of pre-determined sets of cells in Area 1 (the primary auditory cortex, labelled A1 in Fig. 3a) and Area 6 (primary motor cortex, M1 in Fig. 3a). The presence of an activity pattern in Area 6 can be thought of as representing the spontaneous motor-cortical activity that one might observe in M1 during the babbling phase (Fry, 1966). The pattern presented as input to Area 1 simulates the cortical activation that would result in A1 from the near-simultaneous perception of the speech sounds generated by the articulatory movements driven by the activity in M1.

The network ‘training’ consisted of the cyclic presentation of four different pairs of patterns. We used input pattern pairs consisting of randomly generated uncorrelated sparse binary configurations; each configuration included 17 active cells (equalling 2.72% of the total number of cells in one area) and 608 inactive cells. In each cycle, one pair was presented continuously to the network for two time-steps, followed by a period of 50 steps during which no input was given and activity was driven by white noise. A different pair, chosen randomly among the other three, would then follow, until each of the four pairs had been presented to the network 5000 times (for a total of 20 × 10^3^ stimulus presentations). Throughout the training (including the period in which no input patterns were present) the weights of all of the links between E-cells were left free to adapt, according to the Hebbian learning rule described in Section 3.1.2.

After the training, we tested the network with a view to revealing the presence and activation properties of HNCs (cell assemblies) that we predicted would emerge for the given auditory-motor pattern pairs. More precisely, for each of the four pairs presented in input, the time-average of the response (output value or ‘firing rate’) of each E-cell in the network was computed and stored. [The time-averages of the output values were actually computed during the training, recording the cell responses as the four patterns were presented to the network for learning.] These averages were used to identify the four HNCs that developed in the network in response to the four input pairs, as follows: an HNC was defined simply as the sub-set of E-cells exhibiting average output above a given threshold *γ* ∈ [0,1] during stimulus presentation (e.g. if *γ* = 0.75, all cells presenting output above 75% of the output of the maximally active cell in their area during stimulus presentation were considered to belong to the active HNC). Using the above functional definition, we then measured (i) average HNC size and specificity for different values of *γ*, and (ii) average HNC ‘excitability’. The input specificity of an HNC (or, equivalently, the amount of ‘cross-talk’ between pairs of HNCs) was quantified by measuring the overlap (number of cells that two HNCs shared) between the HNCs that emerged in the network as a result of learning. The excitability (or ‘pattern reconstruction’ ability) of an HNC measured how easily (what portion of) an HNC became fully active following partial stimulation (i.e. activation of only a sub-set of its component cells). This was estimated by presenting, for four time-steps, only the Area 1 (auditory) component of the four learnt pairs and by measuring, area by area, the average of (i) the induced HNC activity (as a percentage) and (ii) the total portion of HNC cells that had been reactivated by the stimulus. The averages were calculated across eight different networks, each trained with a different set of four random pattern pairs, producing a total of 32 different (stimulus, network) pairs. The results are shown in Section 4.1.

### 3.3. Influences of lexicality and attention

The second part of the testing simulated the modulatory effects of attention on the brain response to meaningful familiar stimuli (words) and senseless unknown speech-like material (pseudowords). Word perception was simulated in the model by stimulating the auditory cortex (Area 1) of the trained network with the well-learnt patterns used for the training (see previous section); pseudoword perception was simulated by presenting new, ‘unknown’ patterns, built as random combinations of sub-parts of learnt patterns (see Appendix B for details).

The network was tested under different conditions simulating different attentional requirements, induced by systematically varying a single parameter in the model, i.e. the strength of the FI (*α*_5_ in Appendix A, see also Section 3.1.3). Thus, we investigated the effects of attention modulation on the timing and magnitude of the responses to familiar vs. unknown speech stimuli (words and pseudoword) by presenting well-learnt and new activation patterns to Area 1, at increasing levels of FI. More precisely, we stimulated the eight networks previously trained by presenting, for four time-steps: (i) the Area 1 (auditory) component only of the respective four well-learnt ‘word’ pattern pairs and (ii) four different ‘pseudoword’ activation patterns to Area 1. We repeated the stimulation at four different levels of FI (0.90, 1.05, 1.20 and 1.25) and measured the total network activity during the following 50 time-steps. The total network activity was calculated as the sum, across the six areas, of the output values (or ‘firing rates’) of all of the E-cells. The averages (across networks and within lexical category) of the responses are reported in Section 4.2.

## 4. Results

In the next two sub-sections we report the results obtained during the simulation of (i) early stages of word learning (see Section 3.2) and (ii) effects of attention on the brain responses to lexical items (see Section 3.3).

### 4.1. Learning of HNCs

**Fig. 5:**
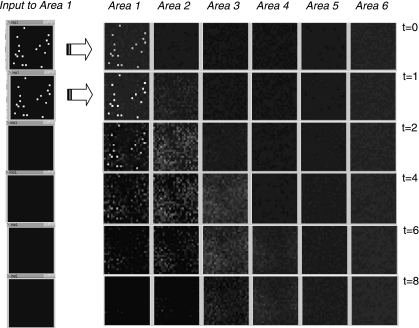
Network response to stimulation of Area 1 (‘auditory’ cortex) with a random activation pattern before training. Each row in the figure is a snapshot (taken at successive time-steps *t* = 0, 1, 2, 4, 6 and 8) of the output activity within each of the six areas (six right columns) and of the pattern presented in input (leftmost column) to Area 1. The different output value (firing rate) of each cell within an area is coded using different brightness levels: very bright or white squares indicate cells with current output ∼1.0, dark or black squares (background) indicate cells with output ∼0.0.

Figure 5 depicts a sequence of six ‘snapshots’ taken at successive time-points and showing the network response to a brief two-step stimulation from the ‘left’ (Area 1 in Fig. 3) with a random activation pattern, before the network has undergone any training. The input given to Area 1 is shown in the leftmost column. In the absence of any training, activity propagates in a rather ‘cloudy’ and unfocused manner, reaching only the first and second areas, and is then dispersed. One point to notice is that the wave of non-specific activation appears to be ‘pushed’ to the right. This is due to the presence of the FI mechanism: the area-specific inhibition loop takes effect as soon as the activation within one area increases and remains active for a few steps; this prevents activation from immediately ‘re-entering’ an area which has just been active.

**Fig. 6:**
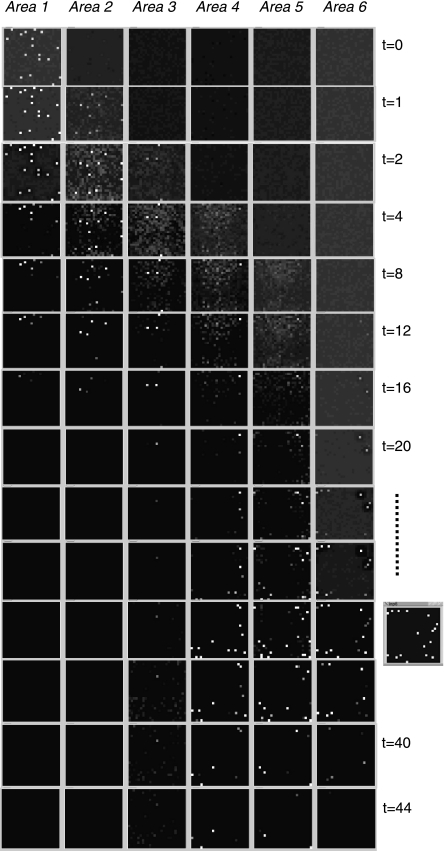
Network response to stimulation of Area 1 with the ‘auditory’ component of one of the learnt pairs after training. Each row is a snapshot of the network output taken at successive time-points (see also Fig. 5). The associated ‘motor’ pattern that the network was trained with is shown, for comparison only, on the righthand side. See text for details.

Figure 6 depicts the result of the network stimulation with an activation pattern after the training has been undertaken. Just like in the previous example, the input pattern (not shown in Fig. 6) was presented to the left (Area 1) for two time-steps only. The response of the network differs significantly from that seen in Fig. 5.

Firstly, notice that, when the input stimulus is no longer present (*t* ≥ 2), if the network is untrained (i.e. the input pattern is ‘unfamiliar’) the cells that had been activated in Area 1 do not remain active for more than one to two time-steps (see Fig. 5); instead, in the trained network, part of these cells remain active for much longer, some even up to 12 steps. This indicates the presence of strong, reciprocal links among these cells in Area 1, learned through Hebbian learning. Secondly, in addition to the propagation of the fast, non-specific wave of activity to the right, the active cells in Area 1 immediately produce strong activation in a specific sub-set of cells in Area 2, already evident at *t* = 1. The activity of these cells is significantly higher than that of cells activated in the surround by the non-specific wave (compare their brightness with that of the active cells in Area 2 of Fig. 5). This indicates that their input must come directly from the strongly active cells in Area 1; hence, these cells receive converging synaptic input from the pattern of active cells presented to Area 1 and respond strongly when this specific pattern is present. Thirdly, as in Area 1, part of these cells remains active well after the ‘cloud’ of activity produced by the non-specific activation wave has disappeared (*t* = 12). This suggests that these cells are also reciprocally connected and project back to some of the strongly active cells in Area 1, creating within- and between-area reverberant activity. The somewhat slower propagation of this activity within specific, isolated cells continues, although the number of cells that are strongly active appears to decrease as the middle and rightmost areas of the network are reached. When the reverberant activity reaches the final area (*t* = 20), an interesting process takes place: from the activity of a few cells situated mostly in the top part of Areas 4–6, an entire new ‘pulse’ of reverberant activation arises, not producing a dispersed cloud but activating very strongly only a specific set of cells in Areas 6, 5 and 4. Notice that when this second slow wave peaks (*t* = 32), the ‘motor’ activation pattern (the single pattern shown in the rightmost column of Fig. 6) that was paired with this specific ‘auditory’ input is reproduced almost entirely in Area 6. Finally, the wave of reverberant activation stops when it fails to activate a specific set of cells in Area 3 strongly enough so as to allow self-sustained activation to continue. This is due in part to the fact that the random and patchy connectivity adopted does not always guarantee the existence of reciprocal links between any pair of cells, and in part to the FI level still being high in Area 3.

The above example begs the question of whether the observed behaviour (which we take as evidence for the presence of distributed cell assemblies strongly associating pairs of ‘sensory–motor’ patterns) is just an isolated case or whether HNCs would emerge in any other network built according to the characteristics described in Section 3.1 and trained with possibly different input pattern pairs. To address this question, we generated and randomly initialized eight different networks and trained each of them with different pairs of random sensory–motor patterns. We then recorded their responses to stimulation of Area 1 only, so as to verify the emergence of HNCs and estimate their average activation properties. The results are described below.

**Fig. 7:**
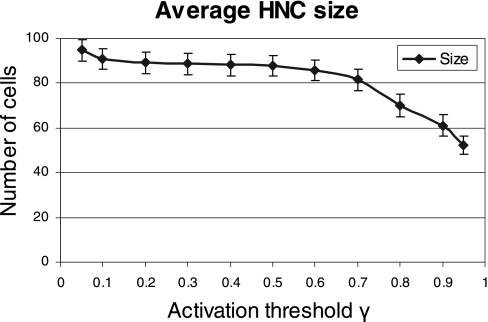
Hebbian neuronal circuit (HNC) size. The average (across eight different networks, each trained with four different input patterns) number of cells within the entire network that responded to a specific input stimulus is plotted as a function of the HNC activation threshold g. For a cell in an area to be counted as a member of the HNC, its average output during a stimulus presentation must be greater than the g portion of the output of the cell that is maximally activated by that stimulus in that area. Vertical bars are SEMs.

Figure 7 plots the average size of an HNC as a function of the threshold *γ*, where an HNC is defined as specified in Section 3.2. As one would expect from such a functional definition, small values of *γ* correspond to larger assembly sizes and vice versa. However, notice that the size of the HNC does not change much when *γ* is in the range [0.05, 0.7] and, even for *γ* = 0.95, the HNC size is still around 50. Thus, the average HNC appears to be a well-identifiable entity formed by a ‘core’ of about 50 cells that respond very strongly (at least 95% of the maximally active cell) to the input stimulus and by an additional ‘belt’ of about 30–40 cells that respond more moderately but still well above average (at least 70% of the maximally active cell).

**Fig. 8:**
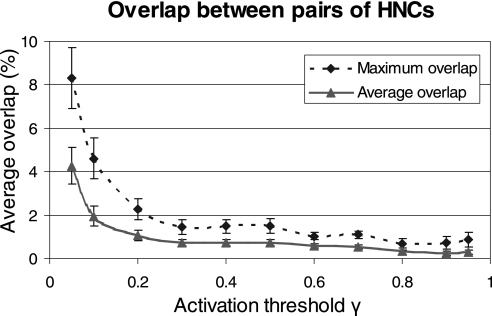
Hebbian neuronal circuit (HNC) distinctiveness. The graph shows the average overlap between pairs of cell assemblies. The maximum (dashed line) and average (solid line) overlaps (in % of cells shared between two HNCs) are plotted as a function of HNC activation threshold g (the average is calculated across eight different networks, each trained with four different pattern pairs). Vertical bars are SEMs.

**Fig. 11:**
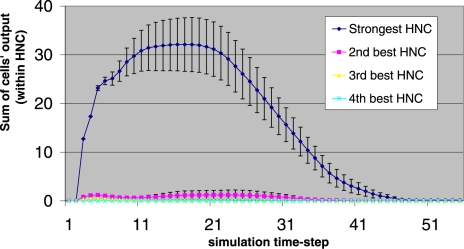
Hebbian neuronal circuit (HNC) specificity. The graph shows the average response of the four different HNCs following ‘auditory’ (layer 1) stimulation with one of the learnt patterns. The total sum of the output (firing rate) of all of the cells within each assembly is plotted as a function of time (in simulation steps). The average is calculated across eight different networks, each network being stimulated (in Area 1) with the ‘auditory’ component of the four learnt stimuli, for a total of 32 network responses. Vertical bars show SEMs. The chosen activation threshold used to identify the cells belonging to an HNC is g = 0.45. FI, feedback inhibition (FI strength = 0.90).

Figure 8 plots the results concerning the HNC distinctiveness. Average and maximum overlap between two HNCs are plotted as a function of the HNC activation threshold *γ*, averaged across eight trained networks. The dashed line plots the maximum overlap (i.e. maximum percentage of cells in an HNC that are shared) between any two HNCs; this is above 5% only for values of *γ* < 0.1. The average overlap between any two HNCs (solid line), however, is always below 5% and less than 2% for *γ* > 0.2. This makes cross-talk very unlikely, as activation of 2–5% of the cells of an HNC is not sufficient to cause full HNC activation (see also Fig. 11).

**Fig. 9:**
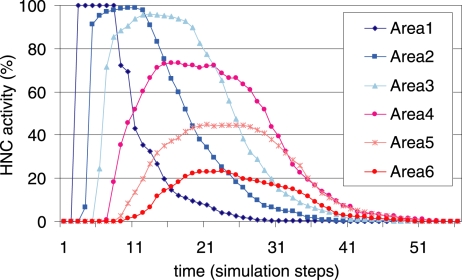
Spatio-temporal pattern of activation of a Hebbian neuronal circuit (HNC). The curves show the average area-specific HNC activation following Area 1 stimulation with one of the learnt auditory patterns (‘words’). The % of HNC cells active above threshold (set here to g = 0.45) is plotted as a function of the simulation time-step. The average is calculated across eight different networks, each trained with four different pattern pairs and stimulated with the ‘auditory’ component only, for a total of 32 network responses. See text for details. FI, feedback inhibition (FI strength = 0.90).

**Fig. 10:**
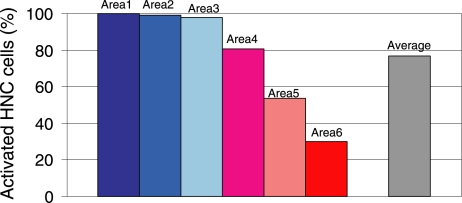
Pattern-completion abilities of a Hebbian neuronal circuit (HNC). The bars show the cumulative portion of an HNC (% of HNC cells per area) that a learnt stimulus presented to Area 1 successfully reactivates over the 50 steps following stimulation, averaged across 32 different ‘auditory’ patterns. These data are calculated by integrating (in time) the curves shown in Fig. 9. The rightmost column is the average of the six area-specific values.

Figures 9 and 10 show the area-specific spatio-temporal activation and pattern completion properties of the HNCs, respectively. Figure 9 plots the percentage of HNC activation in each area produced by stimulation of Area 1 with a learnt ‘auditory’ pattern as a function of time. This percentage is obtained by first specifying an HNC activation threshold *γ* (we used *γ* = 0.45 but, as discussed above, any *γ* ∈ [0.2, 0.7] is expected to produce similar results) that uniquely identifies the HNC cells in each area and then monitoring how many of such cells become active above threshold after stimulation of Area 1 with a learnt activation pattern. The figure delineates how the wave of HNC activation spreads across the network, and the contributions of the different areas to the total activation, each area peaking at a different time and with different intensity.

Figure 10 summarizes the average pattern-completion abilities of the network, plotting the cumulative portion (as a percentage) of HNC cells in the different areas that are reactivated following stimulation of Area 1. This graph is obtained by integrating over time the plots of Fig. 9. As one might expect, pattern completion worsens as activity propagates further away from the input area and activation becomes weaker. The ‘motor’ pattern that had been paired in Area 6 with the (‘auditory’) pattern in Area 1 (now given as input to the network) is, on average, reconstructed only partially (approximately 30%), whereas the average pattern reconstruction across the six areas is above 75%. It should be noted that the network responses to learnt patterns never contained any ‘spurious’ cells; in other words, the only ‘errors’ are missing cells that fail to be fully reactivated. Thus, although the associated pattern is not entirely reconstructed, all of the cells activated by the stimulus are correct and can be seen as a reliable set of ‘core’ representation units.

Finally, Fig. 11 illustrates the results on HNC specificity. The graph shows the average response of the four HNCs when Area 1 is stimulated with one of the four learnt patterns. Each curve plots the sum, across the six areas, of the output (firing rate) of all of the cells of one HNC (again defined by setting *γ* = 0.45) as a function of time (vertical bars show SEMs). HNCs appear to be highly specific; only one HNC is strongly activated by the pattern in input, whereas the others show very little, if any, activity. These results also confirm the conclusions drawn from Fig. 8, which suggested little probability of cross-talk between different HNCs.

### 4.2. Influences of lexicality and attention

**Fig. 12:**
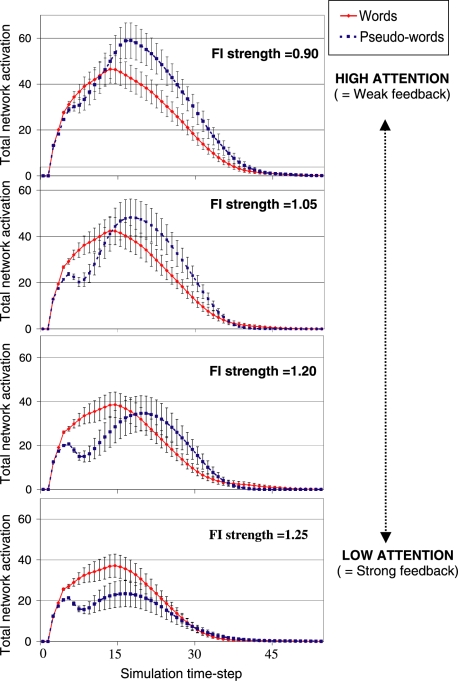
Network simulations of brain response to word (solid lines) and pseudoword (dotted lines) stimuli under different amounts of attentional resources [feedback inhibition (FI) strength]. The total network activation (in abscissa) is computed as the sum of the output values of all of the excitatory cells of the network at a specific time-point. Responses are averaged across eight different networks, which were trained and stimulated with different ‘sensory–motor’ patterns (vertical bars are SEMs). The ‘auditory’ stimulation pattern was presented as input to layer 1 for the period from t = 1 to t = 4. FI strength was varied from 0.90 (top graph) to 1.25 (bottom graph), producing increasing levels of competition between coactivated Hebbian neural circuits and, thus, simulating decreasing amounts of available attentional resources.

Figure 12 shows the results produced by the network when it was used to simulate brain responses to word and pseudoword stimuli under different amounts of attentional resources (see Section 3.3). The graphs plot the total network output as a function of (simulation) time. The dotted and solid curves depict the network response to ‘pseudoword’ and ‘word’ stimuli, respectively. The main point to note is the difference between the top and bottom graphs. In the top graph, weak FI (high attention) produces larger responses to pseudowords than to words, with a ‘late’ peak of the difference between the curves (at 20 simulation time-steps). In the bottom graph, strong FI (low attention) produces the opposite effect (larger responses to words than to pseudowords), with an ‘early’ peaking difference (around 9–10 time-steps). Hence, the modulation of FI strength (or attention) in the network produces a pattern of results that reflects the experimental data discussed in the Introduction (Section 1); in particular, the top graph reflects the characteristics (relative magnitude and latency) of a classical N400 response (see Fig. 1), whereas the bottom graph more closely resembles the features of the MMN response (see graphs in Fig. 2). The mechanisms giving rise to these results are discussed in Section 5.

A second important point to note is that the ‘swap’ in the sign (and change in latency) of the word/pseudoword difference caused by the increase in FI is the result of a strong reduction in the amplitude (and change in shape) of the pseudoword (dotted) curves, and not of an increase in the amplitude of the word response (solid curves). Indeed, if anything, the maximum average amplitude of the word responses appears be attenuated as well, going from about 45 for FI = 0.90 to about 35 for FI = 1.25 (although, looking at the error bars, these curves do not appear to differ significantly from one another).

In sum, these simulation results suggest that the reversed patterns of brain activity observed experimentally under different conditions may be explained by the modulation effects of attention on the cortical responses to pseudoword (and not to word) stimuli.

## 5. Discussion

We implemented a neuroanatomically grounded neural network model of the left-perisylvian language cortex and used it to simulate brain processes of early language learning. We observed the formation of sets of strongly interconnected cells, which we referred to as HNCs or cell assemblies. The trained model was then used to simulate activation of the language cortex when meaningful familiar words (learnt patterns) and senseless unknown pseudowords are presented as input. We found that variation of the amount of area-specific inhibition feedback of the network modulated the relative magnitude and latency of the simulated brain responses to words and pseudowords. More precisely, weak FI (corresponding to high attention and excitability) produced, on average, late activation differences, with a stronger response to pseudowords than to words. This is the response pattern observed in neurophysiological experiments using the N400 component of the ERP as the dependent variable. In contrast, strong FI, simulating suppression and a lack of attentional resources, led to early activation differences, with a stronger response to words than to pseudowords; this closely resembles the relation seen in the MMN data (refer to Figs 1, 2 and 12).

In addition to providing a single unifying framework that explains previously not-well-understood experimental results, the model makes a number of critical predictions (discussed at the end of this section), perhaps the most crucial one being that the brain responses to pseudowords, but not to words, are strongly modulated by attention. Recent MEG experimental evidence (summarized in Section 5.3.1) appears to confirm this prediction.

The discussion that follows is partitioned in three parts, mirroring the structure of Section 3: the first part concerns the neural network model itself; the second part the ability of the network to exhibit spontaneous HNC formation through Hebbian learning; and the last part the simulation of effects of lexicality and attention.

### 5.1. Network structure and function

The neural network model that we implemented mimics the basic properties of the human perisylvian language cortex during word learning and speech stimulation. The basic anatomical properties that were translated into network structure were as follows.

1. The parcellation of perisylvian cortex into M1, PM and PF, and A1, AB and PB, which is known from work in animals and humans.
2. The next-neighbour and long-distance connections linking these areas directly, which is based on work in animals and humans.
3. General principles of cortical connectivity, especially sparseness and patchiness, topography of projections of long-distance connections and next-neighbour preference of local links.
4. Embedding of excitatory cortical neurones into a network of local I-cells.
5. Embedding of excitatory cortical neurones into area-specific inhibitory feedback loops designed to regulate local activation levels.

Although the connections that were implemented in the model are well motivated by neuroanatomical studies in both humans and monkeys (see Pulvermüller, 1992 for a discussion), we only reproduced the predominating next-neighbour connections and long-distance, cortico-cortical links that are known to exist in this part of the brain, and did not include fine-grained details such as connections between non-adjacent cortical areas (e.g. linking A1 to the auditory parabelt). Also, additional areas that might provide additional synaptic ‘stopovers’ on the way from A1 to M1, e.g. in inferior parietal cortex (Catani *et al.*, 2005), were not implemented. There are several reasons for these choices. Firstly, there is little evidence for some of these ‘jumping’ connections. Strong direct connections between primary auditory and motor cortex do not seem to exist. Secondly, neuroanatomical data indicate that each cortical neurone may receive links from fewer than 3% of neurones underlying the surrounding square millimetre of cortex (Stevens, 1989), and that the probability for a connection between two cortical neurones decreases with their distance (Braitenberg & Schüz, 1998). Thus, as a first approximation, we only established next-neighbour and random connections. Thirdly, adding jumping connections (e.g. from Areas 1 to 3 or 2 to 5, as some evidence would suggest, e.g. see Catani *et al.*, 2005) effectively reduces the number of areas that separate area A1 from M1, making the binding of ‘sensory–motor’ pattern pairs even easier and suggesting that similar results should be obtained. Indeed, the model produces analogous results when five, four or three areas are used. This is because a smaller number of areas actually means a shorter path for the ‘auditory’ and ‘motor’ activation patterns to cross during the learning in order to meet the wave of activity coming from the opposite end (in the case of two areas only, the binding between the two input patterns is straightforward and takes place through the reinforcement of the single set of synapses linking the two areas). Hence, the introduction of such more direct links is not expected to produce any significant change in the qualitative behaviour of the network.

An increase in the total number of areas separating the two ‘primary’ areas, A1 and M1, is expected to make HNC formation slower and more difficult. Subject to some parameter changes, however, and up to a certain number of additional areas, results should still hold, although a greater number of training steps will be required. It should be noticed, however, that there is relatively strong evidence for the existence of a six-area pathway connecting A1 to perisylvian primary motor cortex (Pulvermüller, 1992). Even if additional, ‘parallel’ pathways, connecting A1 and M1 through a number of areas higher than six, were introduced in the model (see, e.g. Catani *et al.*, 2005), it is unlikely that their presence would prevent the development of cell assemblies within the shorter, still viable, six-area pathway, which would be automatically used. Finally, we should note that the network is primarily designed as a model of the left language- dominant perisylvian cortex, as the direct links between superior temporal and inferior frontal cortex appear much more developed there than in the non-dominant right hemisphere (Parker *et al.*, 2005). In sum, we think that the present number of six areas constitutes a minimum for approximating the relevant cortical structures and, at the same time, a sufficient level of complexity for replicating and explaining, at cortical-circuit level, the rich dynamics and temporal aspects of the neurophysiological brain responses of interest.

A number of connectionist models of language processing (e.g. Dell, 1986; Norris, 1994; Gaskell *et al.*, 1995; Dell *et al.*, 1999; see Christiansen & Chater, 1999, 2001a,b for reviews) adopt a ‘localist’ approach to the problem, whereby one node of the network does not represent a pool of cortical neurones but a phonological feature, a phoneme or even a whole word. A localist approach would have given us several advantages, including reduced computational load and easier implementation. We chose to make things more difficult (and more realistic) by rejecting one strong assumption made by localist approaches, i.e. that the brain representations of the entities of interest (phonemes, words) and their computational properties are established *a priori*. To clarify, building a (localist or distributed) connectionist model requires specifying, in advance, the computational and representational properties of the nodes of the network. If a node represents a word (or a phoneme), specifying its computational features essentially means to decide, *a priori*, how words (or phonemes) interact and respond to their different input stimuli. Instead, we tried to show and explain how such representations can spontaneously emerge from an initially homogeneous, sparsely and randomly connected brain-like network, through the simulation of well-established neurobiological principles. In the visual domain, the same approach has led, e.g. to the successful modelling of the emergence of ocular dominance and orientation columns in a network with similar connectivity features (Miikkulainen *et al.*, 2005). The cost of adopting this approach, paid in additional complexity and computational load, is offset by two main advantages: (i) the model can be used to simulate and understand the cortical mechanisms that underlie the actual setting up of localist representations (in our specific case, the neural processes underlying early word learning); and (ii) this approach allows us to look at the properties exhibited by the representations that emerge (as opposed to stipulating them as built-in features) and use them to predict the properties of their neural correlates. These predictions (e.g. in our model that the activation of the discrete word representations is relatively unaffected by the current attentional load) can be tested experimentally, providing support for the ability of the model to reflect the relevant brain mechanisms (or evidence to the contrary, thus leading to further refinements of the model and underlying theory).

A second important point is that in most (localist and) distributed connectionist models of language processing (e.g. Seidenberg & McClelland, 1989; Plaut *et al.*, 1996; Joanisse & Seidenberg, 1999; Plaut & Gonnerman, 2000) the learning rule used for simulating synaptic change makes use of information that is not local (i.e. provided by the current activity of the pre- and post-synaptic cells) to the link undergoing the change but obtained from the network's ‘output’ area by means of comparing the current activity in such an area and the desired one (McClelland & Rumelhart, 1986). It is not entirely clear whether (and, if so, how) the brain can actually implement such back-propagating learning mechanisms. We decided to model only neurophysiological mechanisms that are well established and universally accepted (the specific choice of the ABS rule is discussed in the next section).

The present simulation approach is related to a range of recently developed distributed connectionist models that demonstrate how cognitive behaviour emerges from neurobiological structure and function (Tagamets & Horwitz, 1998; Corchs & Deco, 2002; Husain *et al.*, 2004; Deco & Rolls, 2005; Miikkulainen *et al.*, 2005). These models have been used to explain (and simulate PET/fMRI data resulting from) visual and auditory attention phenomena at the mechanistic level of cortical circuits; none of them, however, attempts to address language function.

Most relevant here is the ground-breaking work by Husain *et al.* (2004), who built a neuroanatomically based connectionist model to simulate electrophysiological and fMRI activities in multiple brain regions during an auditory delayed-match-to-sample task for tonal patterns. Their architecture consists of four major brain regions: (i) primary/core auditory cortex; (ii) secondary sensory cortex (belt and parabelt areas); (iii) superior temporal gyrus/sulcus; and (iv) prefrontal cortex. Each region is composed of 81 excitatory/inhibitory units (modified Wilson–Cowan units), each of which represents a cortical column; both feedforward and feedback connections link the different regions. In spite of the apparent similarities, one fundamental difference between this model and the present approach lies in the absence of any learning mechanism. In our model, synaptic plasticity is the (neurobiologically motivated) mechanism that leads to the emergence of the macroscopic phenomena of interest, by means of microscopic interactions taking place in a randomly connected network of identical nodes. In the architecture of Husain *et al.* (2004), different types of cells, exhibiting pre-specified behaviours, are introduced and ‘hard wired’ in an ad-hoc manner. For example, the prefrontal cortex area (see their fig. 1) is assumed to contain four different types of built-in neuronal units: ‘cue-sensitive’ units (assumed to respond when an external stimulus is present), two types of ‘delay’ units (one assumed to be active during stimulus presentation and subsequent delay before presentation of the following stimulus, the other assumed to be only active during the delay between presentations of stimuli) and ‘response’ units, whose activities are assumed to increase when the second stimulus matches the first. These sets of units are assumed to form separate modules, connected by arbitrary links having fixed and pre-determined synaptic weight (Husain *et al*., Table A2). [Note that these built-in properties, especially the active-memory function, have been argued to be the net effect of neuronal assemblies, not a feature intrinsic to single cells (Zipser *et al.*, 1993; Fuster, 2003)]. The secondary area is assumed to contain ‘contour-selective’ units for which there is no direct experimental evidence, and there are no excitatory/excitatory (recurrent) within-area connections in the primary, secondary and superior temporal gyrus/sulcus areas. Finally, the architecture includes an ‘attention’ module [which the authors explicitly declare to be ‘not modelled in a biologically realistic fashion’ (Husain *et al*., p. 1710)] that projects to only one of the two delay modules and directly defines the strength of the representation maintained by such delay units. In spite of these aspects, the architecture of Husain *et al.* (2004) remains, to date, one of the distributed connectionist models of the left perisylvian areas that come closest, in terms of neuroanatomical and neurophysiological realism, to the model presented here.

A recent connectionist model of speech acquisition and production that does incorporate learning mechanisms is described in Guenther *et al.* (2006). This architecture (composed of premotor, motor, auditory and somatosensory cortical areas, in addition to a cerebellum module) is used to simulate a range of acoustic and kinematic data (including compensation to lip and jaw perturbations during speech) and fMRI activity during simple syllable production. The model provides a very effective and insightful account of language processing based on mechanisms that are assumed to simulate neuronal and synaptic level phenomena. To achieve high effectiveness at the functional level whilst maintaining a sufficiently fine-grained level of modelling, however, engineering considerations were prioritized in the implementation at the expenses of neurobiological realism. For example, all projections between the different cortical areas are assumed to be unidirectional (e.g. premotor cortex projects to superior temporal cortex but no projections exist in the opposite direction) and do not exhibit next-neighbour, random and sparse topology [as typically found in the mammalian cortex (Amir *et al.*, 1993; Douglas & Martin, 2004)] but all-to-all connectivity, which is not neurobiologically realistic (Braitenberg & Schüz, 1998; Braitenberg, 2001, p. 63). The model also makes use of some simplifying localist assumptions, e.g. each single cell in the ‘speech sound map’ module [modelling the left ventral premotor cortex (fig. 1 of Guenther *et al*., 2006)] is assumed to represent one specific speech sound, defined as ‘a phoneme, syllable, word, or short phrase that is frequently encountered in the native language and therefore has associated with it a stored motor program for its production’ (Guenther *et al*., 2006, p. 283). In the language acquisition simulation described, a single cell in premotor cortex was used to represent the entire phrase ‘good doggie’. Finally, the tuning of the synaptic weights during the simulation of language acquisition (including the preliminary babbling and subsequent ‘practice phase’, involving the learning of more complex speech sounds) is not realized, like in our model, via a uniform, constantly acting mechanism that closely replicates neurophysiological features of synaptic plasticity and is applied equally to all areas of the model during the training, but through a set of different, ad-hoc procedures of little biological plausibility that are carried out at different times on different sets of synaptic projections. [For example, the synaptic weights of the projections from ventral premotor cortex to superior temporal cortex (‘auditory error map’), encoding the auditory targets for each speech-sound cell, are conveniently ordered in ‘spatio-temporal’ matrices, in which each column represents the target at one point in time, and there is a different column for every 1 ms of the duration of the speech sound. Using an audio file containing the appropriate speech sound, a specified procedure sets up the synaptic weights in such a way that the values are (exactly) the upper and lower bounds of each of the first three formant frequencies, at 1 ms intervals for the duration of the utterance. This ‘learning’ procedure is run once, during the practice phase only (and not during the babbling). However, the weight matrix encoding the projections from premotor to somatosensory cortex is updated only during correct self-reproductions of the corresponding speech sound (i.e. strictly after the learning of the auditory target for the sound). Moreover, in order to account for temporal delays, this process involves artificially aligning the somatosensory error ‘data slice’ with the appropriate time slices of the weight matrices (Guenther *et al*., 2006, their Appendix B).]

Whereas the above models were used to simulate PET and fMRI data, the modelling of EEG/MEG signals has also been the object of research for several years, e.g. epileptic-like (Jansen & Rit, 1995; Wendling *et al.*, 2000), gamma-rhythm (Jefferys *et al.*, 1996) and alpha-rhythm dynamics (Suffczynski *et al.*, 2001) and spectral activity in different frequencies (David & Friston, 2003) have been successfully simulated in the past. However, we are not aware, at present, of any biologically realistic model able to simulate and explain the MEG/EEG dynamics observed during higher-level cognitive and language tasks.

The model presented here, of course, is not exempt from limitations. For example, it does not account for psycholinguistics phenomena related to word frequency, similarity or meaning. However, it is worth noticing that previous work (Wermter & Elshaw, 2003; Wermter *et al.*, 2004, 2005) uses Pulvermüller's model of language and action processing (Pulvermüller, 2001, 2005) implemented as Kohonen maps to simulate processing of words and the actions semantically linked to them. Knoblauch & Pulvermüller (2005) link a neuronal word representation to a syntax network and Knoblauch *et al.* (2005) offer a model of phonological, syntactic and semantic processing in which different areas are implemented as associative memories each dedicated to one linguistic semantic feature.

In summary, many issues still remain to be addressed; the present work focuses on lexicality and attention, and represents only the first step towards the implementation of a large-scale model, envisaged to include visual, somatosensory and motor-cortical modules and to simulate a richer variety of neurophysiological and cognitive phenomena inherent to language.

### 5.2. Learning HNCs

The results shown in Section 4.1 confirm the emergence of HNCs in the network. In particular, each pair of sensory–motor patterns that was repeatedly presented to the network producing simultaneous activation of Areas 1 and 6 led to the formation (through Hebbian learning processes) of a corresponding HNC that associated the ‘sensory’ and ‘motor’ components of the pair. The successful setup of Hebbian circuits spanning a realistic number of cortical areas forming the substrate of perception-action learning is remarkable, as it is commonly believed (O'Reilly, 1998) that learning associations between more than two areas require supervised mechanisms analogous to back-propagation (McClelland & Rumelhart, 1986). Our study, however, provides evidence to the contrary; Hebbian learning was sufficient to learn HNCs distributed over six areas.

The HNCs appear to be strongly interconnected sets of cells that exhibit various properties, including: (i) distributedness and sparseness (Fig. 7) (one HNC consists, on average, of less than 100 cells distributed across the six areas; this is equivalent to less than 2.67% of all cells within the network); (ii) reverberation and persistence of activity in the absence of input stimulus (in Fig. 6, up until 44 time-steps) within well-identifiable self-excitatory loops of cells; (iii) relatively stable size for different critical activation thresholds *γ* (Fig. 7); (iv) small overlap and cross-talk between pairs of HNCs (less than 5% on average), and high specificity of response (Figs 8 and 11); and (v) pattern completion abilities (averaged across areas) above 75%, in spite of the sparse and random character of the network connectivity.

These results, taken together with those presented in Section 4.2, indicate that an HNC behaves as a highly specialized, discrete, ‘all-or-nothing’ functional unit which, if stimulated by an input that matches its specific ‘perceptual’ pattern, becomes fully active regardless of the particular state of the network (Hebb, 1949; Braitenberg, 1978; Pulvermüller, 1999). Hence, the macroscopic behaviour of an HNC is non-linear and characterized by a specific activation threshold, very much like a single neurone. In fact, for the positive-feedback loops that form an HNC to be able to ‘drive’ the circuit towards full activation, it is necessary that sufficient activity is captured by them so that the amount of self-generated excitation overcomes the amount of ‘leakage’ and dispersion. If the activity present in the positive-feedback loops exceeds this threshold (the value of which depends on the specific characteristics − strength, reciprocity − of the internal connections of the HNC), the total activity in the HNC does not dissipate but starts to increase and propagate to the rest of the HNC, in a wave-like fashion (see Figs 6 and 9), producing a momentary ‘pulse’ or peak of activation in the entire HNC (see Fig. 11). This surge of activity in the network [sometimes called ‘ignition’ (Braitenberg, 1978)] causes the area-specific inhibition mechanism to take effect, which then subsequently inhibits the HNC and the entire network (overshoot).

Our implementation of Hebbian learning (Eqn 3) is based on the ABS model of LTP and LTD (Artola & Singer, 1993). The choice of this model among other candidates was motivated by several factors: (i) the ABS model is based on experimental evidence (see Section 3.1.2) and closely mirrors well-known neurophysiological phenomena; (ii) it captures some phenomena that are not modelled by other rules; (iii) it does not lead to behaviours that contradict neurobiological evidence, and (iv) it is computationally tractable.

Compared with the original concept of coincidence learning mentioned by Hebb, which allows synaptic modification only as strengthening of connections between two coactive neurones, the ABS rule envisages, in certain cases, the weakening of links; more precisely, whereas co-occurrence of sufficient pre-synaptic activity [*O*(*x*,*t*) ≥ *θ*_pre_] and strong post-synaptic depolarization [*V*(*y*,*t*) ≥ *θ*_+_] leads to a weight increase (LTP), the presence of only one of these conditions leads to a decrease (LTD). Such weakening contrasts with the ever-increasing synaptic weights that are brought about by coincidence of firing; as explained later in this section, the presence of this feature is crucial to prevent unwanted behaviours such as different HNCs ‘merging’ into a single one.

The ABS rule models heterosynaptic LTD by allowing synaptic change at inputs that are themselves inactive, subject to the post-synaptic cell being strongly depolarized. The well-known BCM rule (Bienenstock *et al.*, 1982) is unable to model this phenomenon, as it requires at least some pre-synaptic activity to be present at a synapse for LTD to take place. The covariance rule (Sejnowski, 1977), another widely used (e.g. Westermann & Miranda, 2004) Hebbian rule that we adopted in previous simulations (Garagnani *et al.*, 2007), can, to some extent, model heterosynaptic LTD but it envisages the strengthening of a link between two cells whenever their activities are both above or below average (including the situation when they are both silent). Not surprisingly, the adoption of this rule produced, in our architecture, an increased likelihood of HNCs merging [compare the average overlaps between HNCs plotted in Fig. 8 with those reported in Garagnani *et al.* (2007)]. The use of a BCM-like rule (obtained by setting *θ*_pre_ = 0 in Eqn 2) led to similar results.

Of course, more realistic learning rules than the one that we implemented can be devised. For example, the synaptic change (learning rate) is currently discretized into only two possible levels (± Δ*w*, see Eqn 3). A more refined set of possible values would, however, introduce significant additional computational load and not necessarily lead to better results.

The adoption of a biologically realistic unsupervised learning rule made the formation of relatively stable cell assemblies more difficult than it would have been using supervised (e.g. back-propagation) learning methods, and made it subject to the optimization of various parameters of the network. In particular, preliminary simulations often presented three interdependent problems that hindered the consistent and reproducible formation of HNCs in the network; we called these problems: (i) ‘contact’, (ii) ‘merging’ and (iii) ‘HNC overgrowth’ (or ‘overlearning’) problems. Appendix C describes these issues in more detail and the parameter changes that we applied to address them. Importantly, in the past, some of these problems have been used as arguments against the feasibility of correlation learning and of the Hebbian cell-assembly model. For example, in a useful compendium of such arguments, Milner (1996) wrote: ‘It is difficult […] to understand why the synaptic modification that links neurones to form an assembly fails to involve more and more neurones until the whole brain becomes one immense and useless cell assembly’ (their p. 70) and later ‘Another serious problem is that an assembly of neurones linked by excitatory connections would be inherently unstable and liable to fire out of control at the slightest disturbance’ (their p. 72).

Our model provides evidence that these problems can be overcome. Firstly, the growth of an HNC is limited by the slow but constant competition for shared cells that takes place between different HNCs. To clarify, every time that an HNC is stimulated, the Hebbian learning causes some synapses to strengthen and (as mentioned earlier) others to weaken. As a result, some cells become more strongly connected to an HNC (i.e. more likely to be activated by it) and less to other, inactive, HNCs. Now, if the network were always confronted with only one stimulus, the corresponding HNC would indeed keep growing and take over the entire network. However, during training, the input stimuli alternate continuously (see Section 3.2); each different stimulus excites a different HNC, possibly overlapping with other HNCs. The continuous alternation of different stimuli causes the cells that are shared by the different HNCs to be alternatively bound more strongly into one or the other assembly. If the input stimuli alternate in a balanced way (as done here), the cells in the overlap never become entirely an exclusive part of any of the competing HNCs; rather, they are the object and site of a constant competition in which each of the assemblies is obstructing the growth of the others, producing a state of dynamic equilibrium.

Secondly, regarding the instability of an HNC (and of the network), spontaneous activation of HNCs during periods in which no input was presented did occur, as predicted, due to the background noise present in the network. [This behaviour was not entirely undesired, as it can be interpreted as a model analogue of a ‘spontaneous thought’.] However, whenever this happened, the self-regulation mechanism (FI) started to operate, causing the HNC to be ‘switched off’ soon after its full activation and preparing the ground for the next HNC activation.

One last point concerns the number of (sensory–motor) pattern pairs used to train and test the network, which is very small (four) when compared with the number of words that our brain can store. Implementing a large-scale network capable of storing a realistic number of lexical items, however, was not one of the objectives of this work; our main aim here was to show proof-of-concept simulations that enable explanations of previous experimental evidence and predictions of future findings. For this purpose, it is sufficient to model the acquisition and processing of a limited number of exemplar sensorimotor patterns, lexical items or words.

### 5.3. Explaining the influences of lexicality and attention

As seen earlier (refer to Section 4.2 and Fig. 12), the simulations show that the amount of area-specific inhibition feedback modulates the simulated brain responses to pseudowords (but not to words), so that weak (strong) FI produces late (early) activation differences, with a stronger (weaker) response to pseudowords than to words. This behaviour fits the neurophysiological data discussed in the Introduction (see Section 1 and Figs 1 and 2), as the N400 response presents a late (around 400 ms) difference, with relatively large responses to pseudowords, whereas the MMN exhibits an early (100–250 ms) difference, with a larger response to words. Until now, in spite of being reproduced in numerous studies, this pattern of results could not be explained, at the cortical-circuit level, by any of the existing connectionist models of language processing. We shall now discuss the underlying mechanisms that make the neural network respond in this particular way.

As pointed out in the previous section, the HNCs that emerged for words behave as discrete, ‘all-or-nothing’ functional units which, when stimulated, become fully active regardless of the FI strength. What happens when a pseudoword is presented as input to the ‘auditory’ area of the network? Recall that a pseudoword pattern consists of a combination of different sub-parts of the four word patterns (see Appendix B). Hence, upon presentation of a pseudoword, the cells belonging to the four different HNCs that happen to be present in the pseudoword are activated in Area 1. Thus, one or more (possibly all four) word HNCs may be simultaneously (but partially) stimulated. If the amount of stimulation they receive is sufficient, activity will start to reverberate in one or more of them. However, due to the presence of (area-specific and local) inhibitory circuits, the different HNCs simultaneously activated will start to reciprocally inhibit each other, in a ‘winner-takes-all’ fashion. This transient period of competition can be observed in the total network responses plotted in the graphs (see Fig. 12); in particular, the pseudoword curves (dotted lines) are ‘S’ shaped, i.e. they exhibit a rapid change of convexity that starts to appear at around three simulation time-steps after stimulus onset (barely noticeable for low values of FI). This effect is due to the fact that, during that period, two or more stimulated HNCs are competing, ‘pushing’ each other down and causing a temporary reduction in (or a reduced rate of increase of) the total network output. What happens afterwards depends entirely on the strength of the FI loops.

In the case of weak FI, there is weak competition between the HNCs; thus, the activity in the ‘winning’ HNC (the one that happened to be more strongly activated, or to contain stronger connections) is not significantly affected by the activity of the other HNCs. Hence, after a short period of competition, the winning HNC resumes its progress [this now takes place in complete absence of the input stimulus, which lasts only two steps] towards full activation, reached at around 20 simulation time-steps. Notice that the competition temporarily slows down the wave-like process of HNC activation, making the HNC peak later than it would have if it had been stimulated in isolation. Simultaneously, activity in the non-winning HNCs is suppressed by the FI (driven by the winning HNC activation). However, because of the presence of reverberant loops in the HNC and of the weak FI, this activity does not immediately disappear but is maintained in the HNC(s) for several simulation steps, and it is still present in one (or more) non-winning HNCs when the winning HNC reaches full activation. Hence, at that point, the total network output is the result of the activity of the winning HNC (at its peak) plus the residual activation in the other HNCs. This makes the peak of the response to a pseudoword larger than that to a word; in fact, all of the rest being equal, the total network activation due to one fully active HNC is (on average) smaller than the total activation due to one fully active HNC plus one or more partially active HNCs. The possible psycholinguistic correlate of this computational process may be the activation of several neighbours of a stimulus pseudoword.

Let us now consider the case of strong FI. Soon after stimulation, the HNCs start to compete and inhibit each other; if the level of FI is sufficiently high, the activity captured by the circuits of the HNCs will be suppressed and will not reach the HNC-specific activation thresholds, preventing the HNCs from entering the unstable positive-feedback state that leads to their full activation. In other words, none of the HNCs is able to ‘survive’ the strong competition and to become fully active; as a result, the total network response to a pseudoword, consisting of the sum of the activities produced by the partially active HNCs, remains (on average) below the total response to a word (which generates a full HNC activation in spite of the strong FI). The difference between the two responses develops very early (as soon as the HNCs start to engage in the competitive process) and also peaks early (at around eight time-steps, as a word HNC stimulated in isolation reaches full activation quickly). The possible psycholinguistic correlates of these processes may be, in the case of a pseudoword stimulus, the lack of recognition of any lexical item and, in the case of words, the ability to automatically recognize and respond to familiar items even when heavily distracted. An example of this phenomenon, known as attentional capture (or ‘cocktail party’) effect, is our ability to automatically detect the sound of our own name even under conditions of inattention (Moray, 1959; Wood & Cowan, 1995).

#### 5.3.1. Fit of model predictions and neurophysiological data

Our model simulates the cortical sources that generate electric potentials and magnetic fields at the surface of the head. Therefore, strictly speaking, our predictions and explanations apply at the level of brain activation and not at that of ERPs and event-related fields. However, the differential activation to words and pseudowords revealed by ERPs/event-related fields is also manifest at the level of sources localized in the perisylvian region (e.g. Pulvermüller *et al.*, 2001; Hauk *et al.*, 2006). Thus, when we speak about larger (smaller) words/pseudowords responses or ERPs/event-related fields, we assume that the corresponding sources are also larger (smaller). Other works have adopted the same approach and successfully modelled EEG/MEG signals as the average depolarization of pyramidal cells (e.g. David & Friston, 2003).

Interestingly, the time-course of the simulated peak differences between word and pseudoword responses roughly reflects the one exhibited by experimental data. In fact, in the model, early differences (see Fig. 12, bottom graph) peak at around seven to eight time-steps after stimulus onset (which is at step 2 in all cases), whereas the late differences peak at 18 time-steps after stimulus onset (Fig. 12, top graph). If we assume that the MMN response peaks at about 120 ms after stimulus onset, one Δ*t* in the simulation corresponds to 120/7 ≈ 17 ms, and the simulations predict a late peak (in the presence of attention) at around 18 × 17 = 306 ms. If, however, we work from the assumption that the N400 response peaks at 400 ms, then one Δ*t* corresponds to 400/18 ≈ 22 ms, and the simulations predict an early peak (when attention is directed away) at around 7 × 22 = 154 ms. Although these calculations should be handled with great care as they are the result of simple extrapolations, they do provide some evidence for the ability of the model to make predictions of the correct order of magnitude on the spatio-temporal patterns of cortical activation. In view of the above, one simulation time-step Δ*t* can be considered to correspond approximately to 20 ms.

One point that needs to be clarified concerns the fact that the early differences observed in the network simulations are not obtained using an ‘oddball’ stimulation paradigm, which is normally required to elicit the MMN response. How can we claim to be simulating the MMN if the network is not being stimulated using an oddball paradigm? Firstly, we are currently using the same network model to reproduce, specifically, the MMN response, this time using an oddball stimulation paradigm. Preliminary results indeed indicate that the MMN response can be spontaneously generated by the network (if appropriate parameters are chosen). Secondly, and most importantly, we take the view that the early differences between words and pseudowords are the result of automatic neural mechanisms that are always at work in the brain (Pulvermüller & Shtyrov, 2006). The simulations are not, indeed, aimed at replicating the MMN response *per se* but the brain mechanisms that underlie and govern the activation of memory traces in the cortex and that we believe generate it. Our simulations predict that these mechanisms are such that the early words/pseudowords difference becomes significant when the subjects are distracted and that this should always happen. This theory, which builds upon our computational model, makes a strong prediction, i.e. that attention modulation should be able to bring out both types of responses (early with words up vs. late with pseudowords up) regardless of the stimulation paradigm used. In other words, modulating attention in an oddball stimulation paradigm should make the MMN larger to words in the absence of attention but produce the reverse effect (MMN larger to pseudowords) when subjects are paying attention to the stimuli. Similarly, in a standard speech listening experiment, an early difference should be present when subjects are heavily distracted, with responses larger to words than to pseudowords; this effect should reverse if subjects are instructed to pay attention to the stimuli. Clearly, these hypotheses need further testing; we are currently undertaking MEG experiments in order to test the former of these predictions, i.e. that the relative magnitudes of the MMN response to words/pseudowords should ‘swap over’ when attention is appropriately modulated.

Some evidence supporting the model's prediction that word-evoked brain responses are not modulated by attention (whereas pseudoword ones are) has been recently found by Shtyrov *et al.* (2007). In this study, the level of attention was systematically varied by using an acoustic stimulus detection task, while the MMN responses to a set of phonologically and acoustically balanced words and pseudowords were recorded using a multideviant oddball paradigm. Under non-attend conditions, the word-elicited MMN (peak at ∼120 ms after words could be uniquely recognized) was significantly larger than that to pseudowords. However, when attention was directed towards the stimuli, the word/pseudoword difference in the MMN was no longer significant; moreover, whereas varying attentional load did not change MMNs to words, the first phase of the pseudoword response was significantly enhanced by attention.

## 6. Conclusions

We present here a model that can simulate processes of cognition using neurophysiologically and anatomically grounded networks. The main contributions of this work are the following: (i) the model is the first to explain and reconcile, at the cortical level and by means of a single, unifying account, the opposite neurophysiological patterns observed for the N400 and MMN responses to speech stimuli; (ii) the simulation results demonstrate the viability of purely Hebbian learning for the formation of (sensory–motor) associations in a hierarchical, brain-like, multilayered neural network architecture; and (iii) the model points to the level of area-specific inhibition feedback as a basis for the brain mechanisms of attention and makes strong predictions on how and why this cognitive process modulates the latency and magnitude of event-related brain responses to speech stimuli.

To sum up, this work represents a first step towards gaining a better understanding of the complex neurophysiological mechanisms at work during the execution of high-level cognitive tasks involving language comprehension and attention.

## Abbreviations

ABS: Artola-Bröcher-Singer
BA: Brodman's area
BCM: Bienenstock-Cooper-Munro
E-cell: excitatory cell
EEG: electroencephalography
ERP: event-related potential
FI: feedback inhibition
fMRI: functional magnetic resonance imaging
HNC: Hebbian neuronal circuit
I-cell: inhibitory cell
LTD: long-term depression
LTP: long-term potentiation
MEG: magnetoencephalography
MMN: mismatch negativity
N400: negative component of event-related potential peaking at around 400 ms.

## Appendix A

This appendix presents details of the network model. Figure 4 displays the generic cortical area model. Our simulations use six such areas in sequence with identical structure and dynamics, and mutual connections between adjacent areas (see Fig. 3b). Each area comprises two mutually connected layers of excitatory neurones (E) and inhibitory cells (I). Their dynamics is given by the following equations:

Activity of E-cells

(A1)

Activity of I-cells

(A2)

Adaptation of E-cells

(A3)

Slow global inhibition

(A4)

In Eqns A1–A4, *V*_E_ and *V*_I_ are the membrane potentials of the E-cells and I-cells on a grid, with *x* = (*x*_1_, *x*_2_), 0 ≤ *x*_1_, *x*_2_ < 25 representing one cell location. We use cyclic boundary conditions. The membrane dynamics is modelled by low-pass filters with time constants *τ*_E_ and *τ*_I,_ respectively. The *ϕ*(*x*,*t*) and *ϕ*_S_(*t*) variables represent cell-intrinsic adaptation and area-specific inhibition (see Section 3.1.3), respectively. Their dynamics is also low-pass, with time constants *τ*_A_ and *τ*_S_. Time constants and time *t* are in arbitrary time units. Dynamic equations are integrated using a simple Euler scheme with step size Δt (Press *et al.*, 1992).

Excitatory cells are graded-response neurones with sigmoid output functions (reflecting firing rates) *f* ^E^(*x*,*t*). We identify *f* ^E^[*V*_E_(*x*,*t*) − *ϕ*(*x*,*t*)] with *O*(*x*,*t*) as defined in Eqn 2, where the parameter *θ* in Eqn 2 corresponds now with the space- and time-dependent adaptation variable *ϕ*(*x*,*t*) in Eqns A1–A4. As Eqn A3 shows, *ϕ*(*x*,*t*) computes a gliding average of the output firing rates of the E-cells, such that *ϕ*(*x*,*t*) gets higher the more strongly a cell is activated. *ϕ*(*x*,*t*) in turn affects the rates of the cells suppressively, acting as a cell-intrinsic dynamic threshold (see also Eqn 2, Section 3.1.1). The inhibitory cells (‘interneurones’) are also graded response neurones but have semilinear rate function *f *^I^ such that *f* ^I^(*x*) = *x* if *x* > 0 and *f* ^I^(*x*) = 0 elsewhere. Note that I-cells were not endowed with an adaptation mechanism, consistent with biology; their main task is to control the activity in the E-cell sub-network locally.

The term *ϕ*_S_(*t*) in Eqn A4 is an additional slow inhibitory process (time constant *τ*_S_ >> *τ*_E_) that provides area-specific activity control by inhibiting all E-cells within one area in equal amounts, proportional to the total within-area activity. This has the net effect of allowing a competition between the different functional representations (HNCs or cell assemblies), restricting activity to the most strongly excited ones (see Section 3.1.3).

The *η*(*x*,*t*) in Eqn A1 are further identical and independent Gaussian white noise processes N(0,1) (Kloeden & Platen, 1995) with noise amplitude *σ* set to 1.04.

Symbols ⊗ in Eqns A1–A4 denote spatial convolution with cyclic boundary conditions in order to avoid boundary effects. (In other words, each ‘convolution’ calculates, for each neurone *x*, the scalar product between its input weights − projection kernel – and its pre-synaptic cells' outputs.) Ranges of the connectivity kernels *k*^FF^, *k*^FB^, *k*^REC^ and *k*^INH^ are indicated in Fig. 4, and their features are explained in detail in Section 3.1.3. The inhibitory kernel *k*^INH^ is identical for all I-cells, i.e. a shift-invariant two-dimensional Gaussian with SD of 2 (lattice units, i.e. cells) and amplitude of 0.295. The precise nature of the initialization of the excitatory kernels as well as the learning rule according to which they change over time is described in the main text (Section 3.1).

Inputs *I*_FF_(*x*,*t*) and *I*_FB_(*x*,*t*) in Eqn A1 are from earlier and subsequent areas, respectively. For the second to fifth area they are the fields of firing rates *O*(*x*,*t*) of the E-cells in the previous and subsequent area but for the first and last areas external inputs are provided as 0/1-bit patterns (clamped input currents).

Finally, the factors *α*_*i*_, *i* = 1,…,5, control the relative weight of feedforward, feedback, recurrent, and fast and slow inhibitory synaptic inputs into the excitatory cells. The network function does not depend crucially on the time constants and connection weights as long as stable operation can be guaranteed.

Parameters used were: *τ*_E_ = 2.5, *τ*_I_ = 5, *τ*_A_ = 15, *τ*_S_ = 37, Δ*t* = 0.5, *α*_1_ = *α*_2_ = *α*_3_ = *α*_4_ = 5, *α*_5_ = 0.9, *α*_A_ = 0.026, *σ* = 1.04. During the testing, *α*_5_ (the area-specific inhibition feedback or FI; see Section 4.2) was varied between 0.90 and 1.25 (see Fig. 12).

## Appendix B

The four pseudoword activation patterns are generated by combining sub-parts of the four ‘word’ patterns (recall that these are 25 × 25 squares of binary configurations containing *n* = 17 cells set to ‘1’ and 608 cells set to ‘0’), as follows.

- For all *i* ∈ {1,…,4}, divide word-pattern *w*_*i*_ into 25 sub-patterns of size 5 × 5.
- For all *j* ∈ {1,…,4}, initialize the pseudoword pattern *pw*_*j*_ as empty.
- Let *j* = 1:
  - copy six randomly chosen sub-patterns from each of the four *w*_*i*_ into *pw*_*j*_, so that the original position of each sub-pattern in *w*_*i*_ is preserved in *pw*_*j*_;
  - if the number of active cells in *pw*_*j*_ is > (<) *n*, set to 0 (to 1) a randomly chosen cell in *pw*_*j*_ until pattern *pw*_*j*_ contains exactly *n*‘ones’.
- Repeat steps 1 and 2 for *j* = 2, 3, 4.

In sum, each pseudoword pattern *pw*_*j*_ is made up of 24 squares (sub-patterns) of size 5 × 5 randomly taken from the four word patterns *w*_*i*_; each word pattern *w*_*i*_ contributes the same number of sub-patterns (six) to each *pw*_*j*_ and each sub-pattern in *pw*_*j*_ is located just where it was in the original word. Thus, this algorithm produces pseudoword patterns that preserve part of the original features of the words (there is a high probability that a sub-set of the active cells of each *w*_*i*_ is reproduced in each *pw*_*j*_ and the total number of active cells in each pattern is exactly the same, *n*) while, at the same time, mixing the four words in a random and balanced way.

## Appendix C

In the early simulations, three interdependent problems often prevented HNC formation.

- *Contact*. For the binding between two coactivated patterns in Areas 1 and 6 to occur, it is necessary that the two ‘waves’ of activity produced are strong enough to reach the middle areas (3 and 4); in addition, these two waves must either (a) jointly activate a common set of E-cells or (b) coactivate two disjoint (but loosely connected) sets of E-cells that will, as a consequence, become strongly linked. To put it simply, for HNCs to develop, the two opposite waves of activity have to make ‘contact’ with each other in the middle of the network. This did not always happen, as the way in which activity from the input areas propagated is strongly influenced by the radius of the within- and between-area projections (parameter *ρ* in Eqn 4). In particular, smaller projection sizes cause more ‘focused’ and stronger propagation of activity towards the middle areas; however, if the projection sizes are too small, neither of the ‘meeting’ conditions (a) or (b) above is likely to be satisfied.
- *Merging*. The different HNCs that developed for the four pairs of input patterns often merged together during the training, becoming, in the worst case, a single HNC that responded to any of the four stimuli (see Milner, 1996). This problem was a symptom of the network inability to learn to ‘discriminate’ between input patterns that produced overlapping network activations. For this type of discrimination to take place, the sets of links between two HNCs and their overlap should be gradually weakened (or, at worst, not strengthened).
- *HNC overgrowth*. During the training, if the number of cells in one of the areas that were strongly activated by one of the stimuli exceeded a certain threshold (around 10–15% of the total number of cells in one area), an unstable positive-feedback loop developed, whereby stronger and stronger responses would follow each new presentation of a given stimulus, leading to the ‘overgrowth’ of one of the HNCs. This HNC would rapidly extend and cover most of the network, causing widespread unphysiological states of saturated activation (notice that overgrowth of one HNC often caused merging and vice versa).

In order to solve these not entirely separable problems, we adopted a combination of different strategies. We do not report the full list here but mention only the key features that played an important role in preventing (or making less likely) the occurrence of these issues.

Firstly, the strength of the local and global inhibition (parameters *α*_4_ and *α*_5_ in Appendix A) had to be calibrated so that sufficient activity would reach the middle areas, whilst, at the same time, the total amount of activity allowed within one area at any one time would be limited so as to prevent overgrowth. Secondly, the time constant *τ*_S_ (see Appendix A) of the FI-cells had to be chosen small enough to increase the speed of the inhibitory response, so that even sudden ‘surges’ of activity would be quickly suppressed. Thirdly, the radius *ρ* of the within- and between-area excitatory projections (see Eqn 4) had to be chosen carefully, as large excitatory projections meant a higher probability of overlap between patterns and of their merging into a single HNC but helped preventing overactivation by making activity more ‘dispersed’ and allowed the linking of coactive cells that were normally too far apart to be bound together, increasing the general ‘pattern completion’ ability of the HNCs. Merging was also made less likely by reducing the ‘density’ of the network connectivity (determined by parameters *k* and *σ* in Eqn 4) and by calibrating the different threshold values of the Hebbian learning rule (*θ*_pre_, *θ*_+_ and *θ*_–_ in Eqn 3). Notice that the radius *ρ* and density of the connectivity had to be chosen in conjunction, as they determine the average number of synaptic links of a single E-cell: if the density increases, radius must decrease for the total number of synapses to remain constant and vice versa. Choosing (and maintaining constant) the total number of synapses is important to avoid over-activation.
